# The mosquitoes (Diptera: Culicidae) of Nuevo León, Mexico, with descriptions of two new species

**DOI:** 10.1371/journal.pone.0217694

**Published:** 2019-08-21

**Authors:** Aldo I. Ortega-Morales, Thomas J. Zavortink, Javier Alfonso Garza-Hernández, Quetzaly K. Siller-Rodríguez, Ildefonso Fernández-Salas

**Affiliations:** 1 Departamento de Parasitología, Universidad Autónoma Agraria Antonio Narro Unidad Laguna, Torreón, Coahuila, MÉXICO; 2 Bohart Museum of Entomology, University of California, Davis, California, United States of America; 3 Instututo de Ciencias Biomédicas, Universidad Autónoma de Ciudad Juárez, Ciudad Juárez, Chihuahua, MÉXICO; 4 Facultad de Ciencias Biológicas, Universidad Juárez del estado de Durango, Gómez Palacio, Durango, MÉXICO; 5 Centro de Investigación en Salud Publica, Instituto Nacional de Salud Publica, Tapachula, Chiapas, MÉXICO; Sichuan University, CHINA

## Abstract

To document the diversity and distribution of mosquitoes inhabiting the Mexican state of Nuevo León, collection trips were conducted to all physiographic regions (Grand Northamerican Plains, Coastal Plain of North Gulf, and Sierra Madre Oriental) and subregions across the state. A total of 3,176 specimens were collected. Additionally, we re-examined mosquito specimens in two Mexican entomological collections: The Collection of Insects and Mites of Medical Importance and the Collection of Arthropods of Medical Importance. These represent the two culicid subfamilies Anophelinae and Culicinae, 8 tribes, 12 genera, 25 subgenera, and 64 named species. Of these, 1 tribe, 2 genera, 5 subgenera, and 14 species are new records for the mosquito fauna of Nuevo León. Three undescribed species were collected. Two are described in this study: *Aedes* (*Ochlerotatus*) *amateuri* Ortega & Zavortink n. sp., and *Aedes* (*Protomacleaya*) *lewnielseni* Ortega & Zavortink n. sp. The third belongs to the genus *Wyeomyia*. Twelve species previously recorded from Nuevo León were not collected during this study. Taxonomic notes, new distribution limits, and comments about the medical importance of some species are reported.

## Introduction

Nuevo León is one of the Mexican states that has been best studied in terms of taxonomy and distribution of mosquitoes (Diptera: Culicidae). Since the end of the 1980s, postgraduate degrees in Medical Entomology (masters and doctorates) have been offered at the Autonomous University of Nuevo León, in the city of San Nicolás de los Garza. Prior to the present study, the list of mosquitoes known to occur in Nuevo León included 44 species that were documented in previous studies, mostly investigations by students [1–7]. However, most of these studies were conducted only in urban or suburban regions, neglecting the mountane forests and other wild regions of the state, resulting in an incomplete checklist. For this study we conducted collection trips during dry and rainy seasons in all the physiographical regions (Grand Northamerican Plains, Coastal Plain of North Gulf, and Sierra Madre Oriental) and subregions of Nuevo León. A current checklist of the mosquito species that inhabit Nuevo León state is provided in this paper. Also, biological notes are provided for species that reach their distributional limits within the state. The species collected and recorded in this study and those located in existing entomological collections are listed in Table 1. We report the presence of three new species; two are described: *Aedes* (*Ochlerotatus*) *amateuri* Ortega & Zavortink n. sp. and *Ae*. (*Protomacleaya*) *lewnielseni* Ortega & Zavortink n. sp. One species belonging to the genus *Wyeomyia* is left undescribed until more material becomes available.

**Table 1:** Checklist of the mosquito species that occur in Nuevo León state.

Species	F.R.	P.S
*Anopheles* (*Anopheles*)		
1. *crucians* Wiedemann	VM	ⅴ
2. *eiseni* Coquillett	Pe	X
3. *franciscanus* McCracken	Sa	ⅴ
4. *pseudopunctipennis* Theobald	VM	ⅴ
5. *punctipennis* (Say)	VM	ⅴ
6. *quadrimaculatus* Say	VM	ⅴ
*Anopheles* (*Nyssorhynchus*)		
7. *albimanus* Wiedemann	Qu	ⅴ
*Aedes* (*Aedimorphus*)		
8. *vexans* (Meigen)	So	ⅴ
*Aedes* **(*Georgecraigius*)**		
9. *epactius* Dyar and Knab	DV	ⅴ
*Aedes* **(*Howardina*)**		
10. ***quadrivittatus* (Coquillett)**	**NSR**	ⅴ
*Aedes* **(*Lewnielsenius*)**		
11. ***muelleri* Dyar**	**NSR**	ⅴ
*Aedes* (*Ochlerotatus*)		
12. ***amateuri* Ortega and Zavortink**	**NS**	ⅴ
13. ***bimaculatus* (Coquillett)**	**NSR**	ⅴ
14. *dorsalis* (Meigen)	So	X
15. *dupreei* (Coquillett)	Va	X
16. *nigromaculis* (Ludlow)	Qu	X
17. *scapularis* (Rondani)	HB	ⅴ
18. *sollicitans* (Walker)	Sa	X
19. *taeniorhynchus* (Wiedemann)	DV	ⅴ
20. ***trivittatus* (Coquillett)**	**NSR**	ⅴ
*Aedes* (*Protomacleaya*)		
21. ***amabilis* Schick**	**NSR**	ⅴ
22. *brelandi* Zavortink	ST	ⅴ
23. ***lewnielseni* Ortega and Zavortink**	**NS**	ⅴ
24. *triseriatus* (Say)	DV	ⅴ
25. *zoosophus* Dyar and Knab	DV	ⅴ
*Aedes* (*Stegomyia*)		
26. *aegypti* (Linneaus)	Va	ⅴ
27. *albopictus* (Skuse)	Or	ⅴ
***Haemagogus* (*Haemagogus*)**		
28. ***equinus* Theobald**	**NSR**	ⅴ
*Psorophora* (*Grabhamia*)		
29. *columbiae* (Dyar and Knab)	Qu	ⅴ
30. *discolor* (Coquillett)	Ma	X
31. *signipennis* (Coquillett)	CD	ⅴ
*Psorophora* (*Janthinosoma*)		
32. *cyanescens* (Coquillett)	Qu	ⅴ
33. *ferox* (von Humboldt)	El	ⅴ
*Psorophora* (*Psorophora*)		
34. *ciliata* (Fabricius)	Ma	ⅴ
35. ***cilipes* (Fabricius)**	**NSR**	ⅴ
36. *howardii* Coquillett	DV	X
*Culex* **(*Anoedioporpa*)**		
37. ***restrictor* Dyar and Knab**	**NSR**	ⅴ
*Culex* (*Culex*)		
38. *bidens* Dyar	IM	X
39. ***chidesteri* Dyar**	**NSR**	ⅴ
40. *coronator* Dyar and Knab	Va	ⅴ
41. *declarator* Dyar and Knab	DV	X
42. ***erythrothorax* Dyar**	**NSR**	ⅴ
43. *interrogator* Dyar and Knab	DV	ⅴ
44. *nigripalpus* Theobald	DV	ⅴ
45. *quinquefasciatus* Say	DV	ⅴ
46. *restuans* Theobald	DV	ⅴ
47. *salinarius* Coquillett	DV	ⅴ
48. *stigmatosoma* Dyar	IM	ⅴ
49. *tarsalis* Coquillett	DV	ⅴ
50. *thriambus* Dyar	DV	ⅴ
*Culex* (*Melanoconion*)		
51. *erraticus* (Dyar and Knab)	Va	ⅴ
*Culex* (*Neoculex*)		
52. ***arizonensis* Bohart**	**NSR**	ⅴ
*Lutzia* (*Lutzia*)		
53. *bigoti* (Bellardi)	El	ⅴ
*Culiseta* (*Climacura*)		
54. *melanura* (Coquillett)	El	X
*Culiseta* (*Culiseta*)		
55. *inornata* (Williston)	DV	ⅴ
56. *particeps* (Adams)	DV	ⅴ
*Mansonia* (*Mansonia*)		
57. *dyari* Belkin, Heinemann and Page	Za	ⅴ
58. *titillans* (Walker)	Za	ⅴ
*Orthopodomyia*		
59. *alba* Baker	DV	X
60. ***kummi* Edwards**	**NSR**	ⅴ
***Wyeomyia* (*Wyeomyia*)**		
61. **undescribed species**	**NS**	ⅴ
*Toxorhynchites* (*Lynchiella*)		
62. ***moctezuma* (Dyar and Knab)**	**NSR**	ⅴ
*Uranotaenia* (*Pseudoficalbia*)		
63. *syntheta* Dyar and Shannon	El	X
*Uranotaenia* (*Uranotaenia*)		
64. ***coatzacoalcos* Dyar and Knab**	**NSR**	ⅴ
65. *lowii* Theobald	Za	ⅴ

The previous literature records are abbreviated: CD [36]; Ma [37]; VM [38]; Va [24]; DV [25]; HB [39]; Sa [1]; IM [26]; Qu [2]; So [3]; Or [4]; El [5]; Pe [6]; Za [7]; ST [40]; NSR (New State Record; new records are in bold); NS (New Species) in boldface; F.R. (First Recorded); P.S. (Present Study). In the last column, a checkmark (ⅴ) indicates the species was collected during the present study and an X indicates it was not collected.

## Materials and methods

### Study area

**Fig 1:**
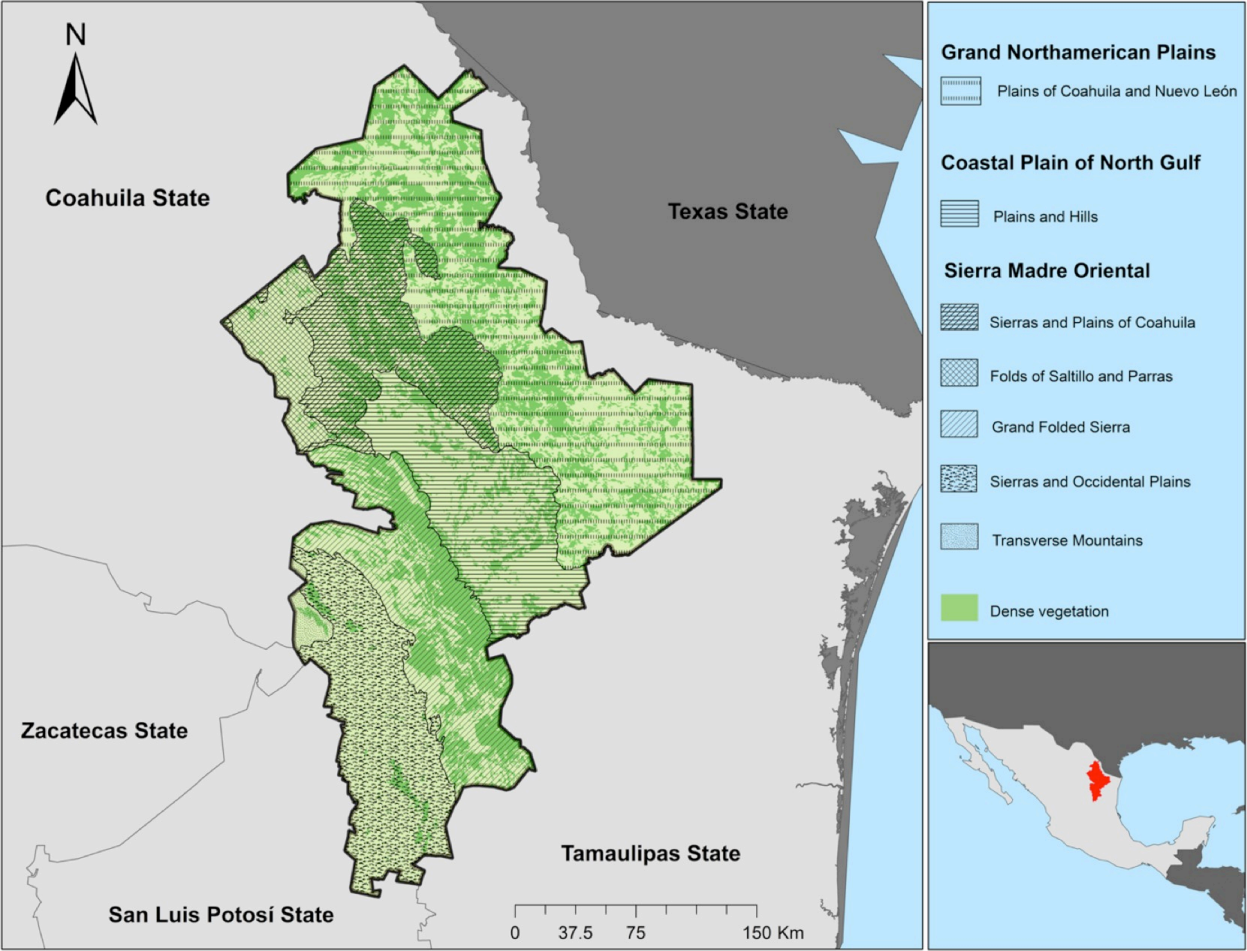
Physiography of Nuevo León state.

Nuevo León state is located in northeastern Mexico, between 23° 10′ 00′′ and 27° 47′ 30′′ north latitude and the meridians 98° 24′ 38′′ and 101° 12′ 9′′ west longitude. The state has an area of 64,924 km^2^. It is bordered to the north by the states of Coahuila, Texas, and Tamaulipas; to the south by the states of San Luis Potosí and Tamaulipas; to the west by the states of San Luis Potosí and Coahuila; and to the east by Tamaulipas. The state is divided into three physiographic regions and seven subregions (Fig 1): Grand Northamerican Plains (Plains of Coahuila and Nuevo León); Coastal Plain of North Gulf (Plains and Hills); and Sierra Madre Oriental (Sierras and Plains of Coahuila, Folds of Saltillo and Parras, Grand Folded Sierra, Sierras and Occidental Plains, and Transverse Mountains) [8]. A description of the regions and subregions of Nuevo León and a list of the municipalities sampled in each are given in Table 2.

**Table 2:** Description of the physiography of Nuevo León state and list of municipalities sampled.

Region (Subregion)	Municipalities sampled	Description of Subregions
**Grand Northamerican Plains (Plains of Coahuila and Nuevo León)**	Agualeguas, Anahuac, Cerralvo, China, Dr. Coss, Gral. Bravo, Gral. Terán, Gral. Treviño, Lampazos del Naranjo, Los Aldamas, Los Herreras, Los Ramones, Melchor Ocampo, Parás, Sabinas Hidalgo, Salinas Victoria, Vallecillo	Only the strip bordering the Rio Grande and the eastern extreme of the subregion. Dominated by hills and plains. The climate is dry and warm in summer, and cold in winter
**Coastal Plain of North Gulf (Plains and Hills)**	Abasolo, Allende, Apodaca, Cadereyta Jiménez, Carmen, Ciénega de Flores, Dr. González, Gral. Escobedo, Gral. Zuazua, Guadalupe, Juárez, Linares, Monterrey, San Nicolás de los Garza	Sandy and clayey soils. The entire subregion is covered by plains interrupted by hills. Dominated by xerophilous vegetation
**Sierra Madre Oriental** (Sierras and Plains of Coahuila)	Bustamante, Hidalgo, Higueras, Pesquería, San Pedro Garza García, Santa Catarina, Villaldama	Located in the northwest of the state, this region includes extensive plains with some isolated low hills. The climate is predominantly arid, the vegetation is xerophilous
**Sierra Madre Oriental (Folds of Saltillo and Parras)**	García, Mina	Occupies a small portion of the west of the state. Includes low mountain elevations. The climate is arid, and the vegetation is xerophilous
**Sierra Madre Oriental (Grand Folded Sierra)**	Aramberry, Gral. Zaragoza, Montemorelos, Santiago	With elevations over 2500 meters. Great vegetational diversity related to climatic variation, from montane forests to tropical forests
**Sierra Madre Oriental (Sierras and Occidental Plains)**	Dr. Arroyo, Galeana, Mier y Noriega	Located to the west of the Grand Folded Sierra, dominated by limestone mountains and soils, generally an arid subregion with xerophilous vegetation
**Sierra Madre Oriental (Transverse Mountains)**	Galeana	Is integrated by perpendicular arid mountains to the Sierra Madre Oriental, with elevations over 2500 meters. The vegetation is xerophilous

### Mosquito collection

Immature stages and adult mosquitoes were collected in specific locations in the three physiographic regions of the state (Table 2). The collections were conducted in both the dry and rainy seasons from 2006 to 2010. Immature stages were collected from all bodies of water encountered. Larvae and pupae were placed in cups with water from the aquatic habitat and transported alive to the Laboraotiorio de Entomología Médica of the Universidad Autónoma de Nuevo León (LEM-UANL) and/or the Laboratorio de Parasitología of Universidad Autónoma Agraria Antonio Narro Unidad Laguna (LP1-UAAAN-UL). A portion of fourth-instar larvae from each collection was mounted on microscope slides using Euparal as the mounting medium, whereas the rest of the live larvae were placed in individual emergence tubes to obtain adults with associated larval and pupal exuviae. Male genitalia were dissected to assist identification when required. Adults were collected in the field using CDC light traps and/or in human biting/landing collectors, and were killed using triethylamine vapor and later mounted on insect pins. Mosquitoes mounted on insect pins were identified using a stereomicroscope Zeiss Discovery V8, while immature stages and exuviae were identified using a microscope Zeiss Primostar. The terminology proposed by Harbach and Knight [9] for mosquito anatomy is used in this study.

### Review of entomological collections

Two entomological collections were reviewed for additional records of mosquitoes from Nuevo León state: the Colección de Insectos y Ácaros de Importancia Médica (IAIM), deposited in the LEM-UANL, and the Colección de Artrópodos de Importancia Médica (CAIM), deposited in the Instituto de Diagnóstico y Referecnia Epidemiológica (InDRE) in Mexico City. The Walter Reed Biosystematics Unit [10] classification for the Culicidae is used in this study.

### Nomenclatural acts

The electronic edition of this article conforms to the requirements of the amended International Code of Zoological Nomenclature, and hence the new name contained herein is available under that Code from the electronic edition of this article. This published work and the nomenclatural acts it contains have been registered in ZooBank, the online registration system for the ICZN. The ZooBank LSIDs (Life Science Identifiers) can be resolved and the associated information viewed through any standard web browser by appending the LSID to the prefix “http://zoobank.org/”. The LSID for this publication is: urn:lsid:zoobank.org:act: 8FDE4640-146E-4E77-8C3D-0B94076E29F1. The electronic edition of this work was published in a journal with an ISSN, and has been archived and is available from the following digital repositories: PubMed Central, LOCKSS.

## Results

**Fig 2:**
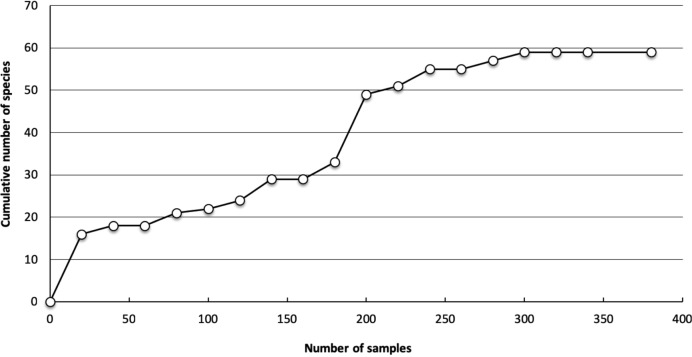
Species accumulation curve for the 58 of the 65 mosquito species (354 collections) collected in Nuevo León during 2006–2010.

A total of 3,176 specimens from 354 collections was studied. Among the specimens were 576 fourth-instar larvae, 420 larval exuviae, 486 pupal exuviae, 50 pupae, 1,105 adult females, 445 adult males, and 94 dissected male genitalia. The mosquito fauna of Nuevo León state consists of 65 species representing the subfamilies Anophelinae and Culicinae, 8 tribes of the subfamily Culicinae, 12 genera, and 25 subgenera (Table 1). One tribe (Sabethini), two genera (*Haemagogus* and *Wyeomyia*), five subgenera (*Georgecraigius*, *Howardina*, *Lewnielsenius*, *Haemagogus*, and *Anoedioporpa*), and 14 species (*Aedes quadrivittatus*, *Ae*. *muelleri*, *Ae*. *bimaculatus*, *Ae*. *trivittatus*, *Ae*. *amabilis*, *Haemagogus equinus*, *Psorophora cilipes*, *Culex restrictor*, *Cx*. *chidesteri*, *Cx*. *erythrothorax*, *Cx*. *arizonensis*, *Orthopodomyia kummi*, *Toxorhynchites moctezuma*, and *Uranotaenia coatzacoalcos*) are recorded from Nuevo León for the first time. Finally, three new species were discovered, two of which (*Ae*. *amateuri* and *Ae*. *lewnielseni*) are described herein. The species accumulation curve of 58 of the 65 mosquito species collected is shown in the Fig 2.

### Biological notes for new state records

#### *Aedes* (*Howardina*) *quadrivittatus* (Coquillett)

Both the subgenus *Howardina* and the species *Ae*. *quadrivittatus* are recorded for the first time in Nuevo León. Females were collected approaching humans in La Camotera, Santiago County, in association with *Ae*. *amateuri*, *Ae*. *trivittatus*, *Ae*. *brelandi*, and *Wyeomyia* n. sp.; biting during the day in association with *Ae*. *muelleri* and *Ae*. *lewnielseni*; and with *Ae*. *brelandi* in oak forest in Santiago County. Immature stages were collected from epiphytic bromeliad axils in Sierra la Camotera, and in La Encantada, Zaragoza County; and from a large tree hole filled with rain water in La Camotera in association with *Ae*. *lewnielseni* and *Cx*. *thriambus*.

#### *Aedes* (*Lewnielsenius*) *muelleri* Dyar

This Nearctic species has been recorded in the United States and Mexico. This is the first record in Nuevo León state for the subgenus and the species. Females were collected biting during the day in La Camotera, Santiago County, in a pine forest in association with *Ae*. *quadrivittatus* and *Ae*. *lewnielseni*; and immature stages were collected in the same location from a tree hole in association with *Cx*. *restrictor*.

#### *Aedes* (*Ochlerotatus*) *bimaculatus* (Coquillett)

This species was collected in only one location in Dulces Nombres, Pesquería County. One female was collected at night in a CDC light trap (baited with CO_2_) located near a pond with abundant emergent and floating vegetation, such as water hyacinths, in a region of shrubs. Associated species were *An*. *pseudopunctipennis*, *An*. *quadrimaculatus*, *An*. *albimanus*, *Ae*. *scapularis*, *Ae*. *taeniorhynchus*, *Ps*. *ciliata*, *Cx*. *coronator*, *Cx*. *quinquefasciatus*, *Cx*. *erraticus*, *Ma*. *titillans*, and *Ur*. *lowii*.

#### *Aedes* (*Ochlerotatus*) *trivittatus* (Coquillett)

Although *Ae*. *trivittatus* is widely distributed in Mexico, this is the first record for Nuevo León state. Females were collected biting humans during the day in oak forest in La Camotera, Santiago County, in association with *Ae*. *quadrivittatus*, *Ae*. *amateuri*, *Ae*. *brelandi*, and *Wyeomyia* n. sp.; in an area of shrubs in Mina County in association with *Ps*. *cyanescens*; and in caves in Lampazos del Naranjo County. Females were collected at night in Lagunita de Taberna, Mier y Noriega County; and in Ciénega de González, Santiago County, in association with *Ae*. *amateuri*, *Ae*. *amabilis*, *Ae*. *triseriatus*/*Ae*. *brelandi*, *Ps*. *ferox*, *Cs*. *particeps*, and *Ur*. *coatzacoalcos*. Specimens were also collected at night in octenol-baited CDC light traps in Lagunita de Taberna; in a CDC light trap without bait in Ciénega de González, in association with *Ae*. *amabilis* and *Cs*. *particpes*; and in a CDC light trap without octenol located in a shrubby area in Las Labores del Ojo, Mina County, in association with *An*. *pseudopunctipennis*, *Ps*. *columbiae*, *Ps*. *signipennis*, and *Ps*. *cyanescens*.

#### *Aedes* (*Protomacleaya*) *amabilis* Schick

The presence of *Ae*. *amabilis* in Nuevo León is the most unexpected record in this study. Since the species was originally described from females collected in Cueva del Nacimiento del Agua, Veracruz state, Mexico, by Schick [11], it has been recorded only in Tamaulipas state. Females were captured biting collectors at dusk in El Salto, Zaragoza County, and biting at night in Ciénega de González, Santiago County. Associated species were *Ae*. *amateuri*, *Ae*. *trivittatus*, *Ae*. *triseriatus*/*brelandi*, *Ps*. *ferox*, and *Cs*. *particeps*. Additional females were collected at night in CDC light traps in the latter location; associated species were *Ae*. *trivittatus* and *Cs*. *particeps*. All of the collection sites were in oak forest. No immature stages of *Ae*. *amabilis* were collected.

#### *Haemagogus* (*Haemagogus*) *equinus* Theobald

The genus *Haemagogus*, subgenus *Haemagogus*, and *Hg*. *equinus* are recorded for the first time in Nuevo León. Females were collected in only one location in Belisario Domínguez, Linares County, during the day in a region of shrubs in association with *Ae*. *aegypti*, *Ae*. *albopictus*, and *Ps*. *ferox*.

#### *Psorophora* (*Psorophora*) *cilipes* (Fabricius)

The presence of *Ps*. *cilipes* in Nuevo León is another unexpected record in the state. This species was collected in only one location in Apodaca County, by aspirating in shaded vegetation in a suburban area near a large pond with abundant emergent vegetation. Dissection of male genitalia was required to identify this species. Associated species were *Ae*. *vexans*, *Ps*. *columbiae*, *Ps*. *cyanescens*, and *Ps*. *ciliata*.

#### *Culex* (*Anoedioporpa*) *restrictor* Dyar and Knab

This species was collected only in a tree hole filled with rain water in an oak forest in Sierra la Camotera, Santiago County, in association with *Ae*. *muelleri*. *Culex restrictor* has been recorded as far north as Tamaulipas, and this is the first report in Nuevo León state.

#### *Culex* (*Culex*) *chidesteri* Dyar

This is one of two species of the subgenus *Culex* that are new records for Nuevo León state. Immature stages were collected from a ground-level water channel in an oak forest in San Juan Bautista, Santiago County, in association of *An*. *franciscanus*, *Cx*. *thriambus*, *Cx*. *restuans*, and *Cs*. *particeps*; and from an artificial container in Belisario Domínguez, Linares County. Adults were collected at night in CDC light traps in Rancho las Flores, Dr. Coss County, in a shrubby area in association with *An*. *pseudopunctipennis* and *Ps*. *cyanescens*.

#### *Culex* (*Culex*) *erythrothorax* Dyar

This is the first report of *Cx*. *erythrothorax* in Nuevo León state, where it was found in just one location in Bustamante County. Females were taken at night using CDC light traps in a shrubby area of Bustamante in association with *An*. *pseudopunctipennis*, *Ae*. *vexans*, *Cx*. *coronator*, and *Cx*. *tarsalis*; and in the same locality where females were collected biting humans at night in association with *Ae*. *vexans*, *Ps*. *signipennis*, and *Cx*. *coronator*.

#### *Culex* (*Neoculex*) *arizonensis* Bohart

This is the first report of *Cx*. *arizonensis* and the first record for the subgenus *Neoculex* in the state. Immature stages were collected from discarded tires with colored water in La Encantada, Zaragoza County, in association with *Cx*. *thriambus* and *Cs*. *particeps*; from a rock hole with colored water in Lagunita de Taberna, Mier y Noriega County; and from springs in La Camotera, Santiago County, in association with *An*. *pseudopunctipennis* and *Cs*. *particeps*.

#### *Orthopodomyia kummi* (Coquillett)

In Mexico, the genus *Orthopodomyia* is represented by *Or*. *alba* Baker, *Or*. *kummi*, and *Or*. *signifera* (Coquillett). This is the first record of *Or*. *kummi* in Nuevo León state. Females were collected at night in CO_2_-baited CDC light traps located in oak forest in Rincon de la Silla, Guadalupe County; adults were found resting in vegetation in La Camotera, Santiago Coiunty; and immatures were collected from a tree hole filled with rain water and abundant litter in oak forest.

#### *Wyeomyia* (*Wyeomyia*) n. sp.

The genus *Wyeomyia* includes 13 species in Mexico, mostly distributed in the southeastern region of the country where tropical forests are located. Two species occur in the Nearctic forest of northeastern Mexico: *Wy*. *mitchellii* (Theobald) is known from Biosphere Reserve El Cielo, in Tamaulipas state, and this undescribed species collected in Nuevo León in La Camotera, Santiago County. Two females of the new species were collected biting humans at dusk in oak forest in association with *Ae*. *quadrivittatus*, *Ae*. *amateuri*, *Ae*. *trivittatus*, and *Ae*. *brelandi*. These specimens are in poor condition; hence, this species will be formally described when more speciemens, including immature stages and adult males, are obtained.

#### *Toxorhynchites* (*Lynchiella*) *moctezuma* (Dyar and Knab)

Prior to this study, species of the genus *Toxorhynchites* recorded Nuevo León by a number of authors were identified as *Tx*. *rutilus* (Coquillett), *Tx*. *theobaldi* (Dyar and Knab), and *Toxorhynchites* sp. Since the identity of the species of *Toxorhynchites* in northern Mexico is still uncertain, the species recorded here as *Tx*. *moctezuma* is based on studies by Zavortink and Chaverri [12]. *Toxorhynchites moctezuma* is thus a new state record for Nuevo León. Adults were collected resting in vegetation in a shrubby area in Ampliación Nogales, García County, in association with *Ae*. *aegypti* and *Ae*. *albopictus*; approaching humans in the same location in association with *Ae*. *scapularis* and *Ae*. *albopictus*; and resting in vegetation in Allende County in association with *Cx*. *coronator* and *Cx*. *quinquefasciatus*. Immature stages were collected from a water tank with clear water in Rancho la Primavera, Santiago County, in association with *Ae*. *albopictus*, *Cx*. *coronator*, and *Cx*. *quinquefasciatus*; from a discarded tire with colored water in Unidad Piloto, Guadalupe County; from a variety of artificial containers, such as plastic buckets and discarded tires, in Pesquería County in association with *Ae*. *aegypti*, *Ae*. *albopictus*, *Cx*. *coronator*, and *Cx*. *quinquefasciatus*; from a tree hole in Bio-Parque Estrella, Montemorelos County, in association with *Ae*. *epactius* and *Ae*. *albopictus*; from a plastic bucket with colored water in San Juan Bautista, Santiago County, in association with *Ae*. *albopictus*, *Cx*. *quinquefasciatus*, *Cx*. *thriambus*, and *Ur*. *coatzacoalcos*; from a discarded tire in Linares County; from a tree hole in a semi-arid area in El Potrero, Villaldama County, in association with *Ae*. *albopictus*; and from a plastic bucket at the last location.

#### *Uranotaenia* (*Uranotaenia*) *coatzacoalcos* Dyar and Knab

This species is recorded for the first time in Nuevo León state. Immature stages were collected from an artificial container (plastic bucket) filled with rain water and abundant litter that colored the water. The specimens were collected in San Juan Bautista, Santiago County; associated species were *Ae*. *albopictus*, *Cx*. *quinquefasciatus*, *Cx*. *thriambus*, and *Tx*. *moctezuma*. Adults were collected approaching humans at night in Ciénega de González, Santiago County, in oak forest in association with *Ae*. *amateuri and Ae*. *trivittatus*.

### Descriptions of new species

#### *Aedes* (*Ochlerotatus*) *amateuri* Ortega and Zavortink n. sp. 4D562DD9-C4E7-4224-9CE6-4E6E12868149

Type specimens: *Holotype*: adult female (A♀) without associated larval and pupal exuviae [CC-UL, 01110805-SAN], El Manzano, Santiago, Nuevo León, Mexico (25° 22′ 24.5′′ N 100°13′11.14′′ W), elevation 1,364 m, immature stages collected from a small pond with colored water without aquatic vegetation, 11 Aug 2005, col. A.I. Ortega-Morales. *Paratypes*: 1A♀, (same data as *holotype*); 7 fourth-instar larvae [CC-UL, 01090507-CC], 5 fourth-instar larvae [Bohart Museum], 1 larval exuviae with dead pupa [Bohart Museum], 1 pupal exuviae (Pe) with A♀ [Bohart Museum] (1–102), 1 larval exuviae (Le) with Pe, and A♀ (charola 1) [Bohart Museum], Cola de Caballo, Santiago, Nuevo León, Mexico (25° 22′ 5.3′′ N 100° 9′ 29.5′′ W) elevation 556 m, immature stages collected from a small pond, 9 May 2007, col. A.E. Elizondo-Quiroga, A.I. Ortega-Morales; 10A♀ [CC-UL, 01200610-CG], 5A♀ [Bohart Museum], Cienega de González, Nuevo León, Mexico (25° 22′ 39′′ N 100° 14′ 31′′ W), elevation 1,331 m, biting collectors, 20 Jun 2010, col. J. Rodríguez-Rojas; 4A♀ [CC-UL, 02120610-EM], El Milagro, Cruillas, Tamaulipas, Mexico (24° 40′ 41.5′′ N 98° 38′ 2.4′′ W), elevation 210 m, biting collectors, 12 Jun 2010, col. A. Hernández-Hernández, A. Sánchez-Trinidad, F. Ordóñez-Sánchez, O. Mandujano-Grajales; 4A♀ [CC-UL, 07120610-SSC], 2A♀ [Bohart Museum], Sierra de San Carlos, Cruillas, Tamaulipas, Mexico (24° 39′ 32.9′′ N 98° 36′ 46.5′′ W), elevation 210 m, biting collectors, 12 Jun 2010, col. A.I. Ortega-Morales, A. Sánchez-Trinidad, O. Mandujano-Grajales.

**Fig 3:**
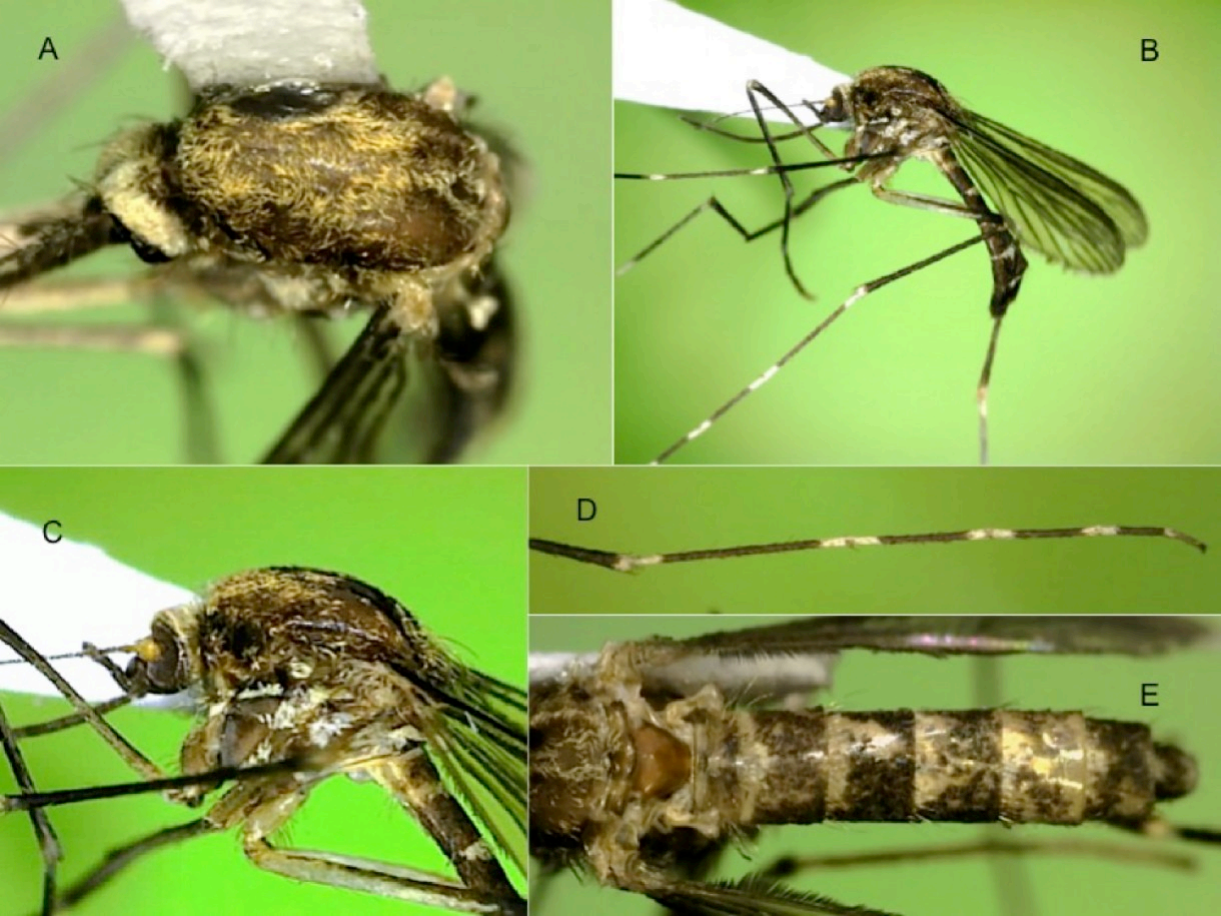
Adult female of *Aedes* (*Ochlerotatus*) *amateuri*. A: Dorsal view of the scutum. B: Laterial view of the body. C: Lateral view of thorax. D: Lateral view of foreleg. E: Dorsal view of abdomen.

Female. Wing: about 3.79–3.84 mm. Proboscis: about 2.0–2.30 mm. Forefemur: about 1.87–1.94 mm. Abdomen: about 3.0–3.3 mm. Integument brown to light brown, thoracic pleura sometimes lighter brown. *Head*: Eyes moderately separated above antennae, interocular space with several light-colored setae and narrow curved white scales. Occiput with erect scales broad, creamy-white near midline, usually becoming tan or brown laterally. Vertex with narrow curved creamy-white scales along midline and eye margin and broad flat creamy-tan to light brown scales on dorsolateral, lateral and ventral surfaces, the darkest scales usually dorsolateral. Proboscis 1.1–1.2 length of forefemur, dark-scaled. Palpus about 0.2 length of proboscis, with 3 obvious palpomeres, dark-scaled. Antenna subequal to proboscis in length; pedicel usually yellow, darker and with a few light or dark scales mesally; flagellomere 1 slightly swollen, with white scales mesally. *Thorax* (Fig 3A): Acrostichal setae few, on anterior area of scutum; dorsocentral setae few, on anterior and posterior areas of scutum; prescutellar and supraalar setae numerous; humeral setae 2, 3. Scutellum with 10–12 setae on each lobe. Scutal scales narrow, curved, whitish, dingy white, or cream-colored on anteriorly, around prescutellar space and in antealar area, yellowish in diffuse narrow acrostichal and broader outer dorsocentral lines, light brown to light reddish brown in diffuse inner dorsocentral line, and dark reddish brown laterally from anterior promontory to above wing base. Scutellum with numerous narrow curved cream-colored scales, those on lateral lobe sometimes yellowish to brown. Paratergite without scales (Fig 3B). Antepronotum with setae and brown and/or cream-colored narrow curved scales. Postpronotum with vertical row of setae posteriorly, narrow curved mostly brown scales dorsally, and broad flat white scales posterioventrally. Pleura with light-colored setae on postspiracular area, prealar knob, proepisternum, upper and posterior mesokatepisternum and upper mesepimeron; with broad flat mostly appressed white scales on postspiracular and subspiracular areas, the latter patch with dorsal extension to postpronotum, lower proepisternum, proepisternum, in patch on prealar knob below setae, in upper patch on mesokatepisternum that does not reach anterodorsal corner of sclerite, in lower patch along posterior margin of mesokatepisternum, and in large, somewhat divided patch on upper 0.67 of mesepimeron (Fig 3C). *Wing*: Completely dark-scaled. *Halter*: Light-scaled. *Legs* (Fig 3D): All coxae with broad flat white scales on anterior surface, forecoxa usually with additional dark scales. All femora with white-scaled knee spot also involving base of tibia; fore- and midfemora predominantly dark-scaled, white scales on posterior and ventral surfaces; hindfemur mostly white-scaled with dorsal dark-scaled line beginning near base and broadening distally to become an apical dark-scaled band. Tibiae each with narrow incomplete apical white band. Fore- and midtarsus with white scales in complete narrow band at base of tarsomere 1, in complete to incomplete narrow band over joint between tarsomeres 1 and 2, and usually in inconspicuous incomplete bands over remaining joints or at the base of tarsomeres 3–5; hindtarsus with white scales in conspicuous complete band at base of tarsomere 1, in bands over joints, and completely covering tarsomere 5. *Abdomen* (Fig 3E): Tergum I entirely or mostly dark scaled dorsally, bare or with a few scales laterally; laterotergite bare. Terga II–VII with white scales in basolateral patches and separated narrow to broad basal bands usually broadest in middle; terga V–VII or VI, VII with light scales at apex. Sterna white scaled, III–VII sometimes with dark-scales in small apicolateral patch.

Male. In general, as in female except for sexual characters and scutal ornamentation. *Head*: Proboscis about 1.15 length forefemur. Palpus long, with 5 palpomeres, 2 and 3 ankylosed and long, 4 about 0.17 and 5 about 0.15 length of palpus; apex of palpomere 3 slightly swollen, upturned and with long setae on underside; palpomeres 4 and 5 usually slightly downturned, 4 with long setae on underside, 5 with shorter setae on underside; dark-scaled except inconspicuous light-scaled patch at base of 5 dorsally. Antenna short, extending to apex of palpomere 3, flagellomeres 12 and 13 comprising 0.5 length of flagellum. *Thorax*: Scutal scale-pattern not as distinct as in female, light scales more extensive, sometimes without brownish scales in dorsocentral line. *Legs*: Fore- and midtibiae often with posterior streak of pale scales. Anterior fore- and midclaws greatly enlarged, with blunt submedian tooth and acute basal tooth; posterior fore- and midclaws moderately enlarged, with acute subbasal tooth; hindclaws small, with acute submedian tooth.

**Fig 4:**
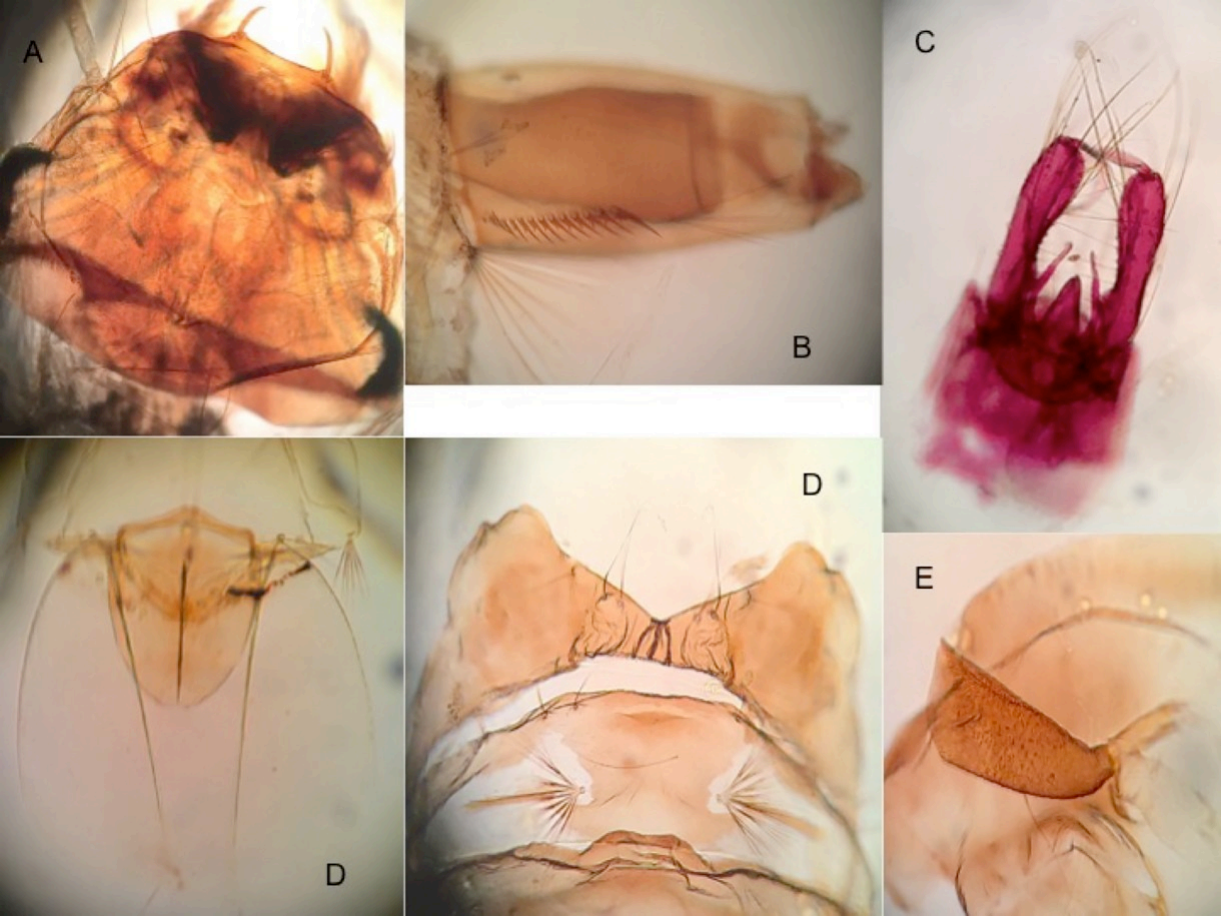
Male genitalia, pupa, and larvae of *Aedes* (*Ochlerotatus*) *amateuri*. A: General aspect of genitalia. B: Trumpet. C: Metanotum and seta 1-I. D: Paddle with genital lobe (male). E: Head (dorsal view). F: Pecten of siphon.

Male genitalia (Fig 4A). *Segment IX*: Tergum well developed, with narrow, deep emargination between prominent tergal lobes, the lobes slightly broader than long, bearing 5, 6 short, strong setae; sternum with 2 setae distally. *Gonocoxite*: Length about 3.6 times median width; basal tergomesal lobe well developed, bluntly conical, with 1 very strong, bent and recurved dorsal differentiated seta, about 20 weak setae, and 3 or 4 slightly longer and stronger setae along ventral margin; apical tergomesal lobe weakly developed, with short setae; tergal surface with numerous short setae and with 2, 3 long, strong setae distad; lateral surface with numerous long, strong setae and scales; sternal surface with numerous moderate to strong setae; sternomesal margin with long row of 25–30 weak to moderate setae; mesal surface membranous. *Claspette*: Stem elongate, extending well beyond basal tergomesal lobe, curved dorsad, with tubercle near base of ventral surface bearing single small seta; filament subequal in length to stem, expanded near middle, without retrorse process, with slender, curved, acute apex. *Gonostylus*: Simple, long, slightly expanded before mid-lenght, curved inward distally, with inconspicuous spicules on dorsal surface; spiniform long, slender, about 10–12 times width, and 0.25 length of gonostylus. *Phallosome*: Aedeagus small, more or less ovate, with slight apical emargination. *Proctiger*: Paraproct strongly sclerotized, with single, large, strong, apical tooth; cercal setae small, 3.

Pupa. Abdomen: about 2–5–2.9 mm. Trumpet: about 0.45–0.47 mm. Paddle: about 0.59–0.65 mm. With typical countershading, the dorsal portions in life very light brown, the ventral areas straw-colored to nearly colorless. *Cephalothorax*: Seta 6-C weakly developed, remaining setae moderately developed, most setae single to few branched or few-forked; setae 8,9-C single or 2-forked; seta 10-C with 6–8 branches. *Trumpet* (Fig 4B): Uniformly brown, broadening gradually from base to apex, pinna small. *Abdomen*: Seta 1-II moderately strong, with 13–21 branches, closer to midline than seta 1-I (Fig 4C); seta 1-IV–VII weakly to moderately developed, usually double (single to triple or 2-forked). Seta 2-II inserted laterad of seta 3-II; seta 2-III–VII inserted mesad of seta 1 of corresponding segment. Seta 3-II single; seta 3-III moderately strong, single, shorter than length of tergum IV; seta 3-V–VII usually single (single or double). Seta 5-IV–VI strongly developed, longer than succeeding segment on IV, V, shorter on VI, single, double, or 2-forked; 5-VII weakly developed, shorter than 3-VII, with 2–4 branches. Seta 6-I,II moderately long, fine; seta 6-III–VI weakly to moderately developed, single; seta 6-VII with 4–6 branches. Seta 9-I–VI small, single; seta 9-VII moderately developed, double or triple; seta 9-VIII strongly developed, dendritic, with 6–9 main branches. *Terminal segments* (Fig 4D): Male genital lobe 1.2 times length of tergum VIII. *Paddle*: Very lightly pigmented, longer than broad, index 1.1–1.3, apex weakly emarginate. Midrib not extending to apex; external buttress weakly developed, indistinct, very weakly serrated. Seta 1-P long, single.

Larva. Head (Fig 4E): about 1.00–1.05 mm. Siphon: about 0.60–0.64 mm. Saddle: about 0.22–0.28 mm. *Head*: Broader than long, moderately pigmented, lighter in ocular area, darker posteriorly. Seta 1-C stout. Seta 4-C weakly developed, about 0.25 length of seta 6-C, 2–4 forked, inserted mesocaudad of seta 6-C. Seta 5,6-C single, nearly equidistant from midline of head, inserted laterad of 1-C. Seta 7-C with 4–6 branches. Seta 15-C short, with 2–5 branches. Dorsomentum triangular, with 12–15 teeth on each side of median tooth. *Antenna*: Short, less than 0.5 length of head, weakly pigmented, slightly darker beyond middle, weakly and sparsely spiculose. Seta 1-A arising near middle of shaft, not extending to apex, with 2–5 branches. *Thorax*: Without conspicuous pigmentation. Spicules of thoracic integument numerous, weakly pigmented, length 4–6 times basal diameter. Tracheal trunks conspicuously dilated in metathorax. Seta 1–6-P single, setae 2,3-P shorter and weaker than seta 1-P. Seta 7-P usually triple (double, triple). Seta 1-M single, less than 0.5 length seta 5-C. *Abdomen*: Seta 1-III with 2–4 branches; seta 1-IV,V strongly developed, long, single. Seta 6-I,II usually double (double or triple); seta 6-III–VI single. Seta 8-II single, double. Seta 13-II,VI short, multiple, subequal; seta 13-III–V strongly developed, long, single. *Segment VIII*: Comb scales small, evenly fringed, about 19–26 in number, in small patch of 2 or 3 irregular rows. *Siphon*: Moderately pigmented, with darker basal band; short, index 2.2–2.5; acus distinct, attached. Pecten spines teeth dark, 12–18 in number, progressively longer distad, those in distal half of row with 1 large and 2 or more basal denticles; evenly spaced, in straight row extending to middle of siphon (Fig 4F). Seta 1-S inserted slightly distad of and in line with pecten, with 5–8 branches, shorter than basal diameter of siphon, slightly shorter and weaker than seta 5-VIII. Seta 2-S small, inserted near apex of siphon. Accessory dorsolateral setae absent. *Anal segment*: Saddle completely encircles segment, moderately pigmented, with darker basal band; acus distinct, attached; without caudal marginal spicules. Seta 1-X arising on and shorter than saddle, single. Seta 2-X with 6–9 branches, less than 0.5 length seta 3-X. Seta 3-X single, long. Ventral brush (seta 4-X) strongly developed, with 14–17 seta on strongly developed grid; no precratal setae. Anal papillae pointed, dorsal papillae 2.4–3.0 length of anal saddle, ventral papillae slightly shorter.

Systematics. From its initial recognition by Dyar [13–14] until the present time, the Scapularis Group of *Aedes* has usually been interpreted to consist only of species with dark, unbanded tarsi. Arnell [15], noted that the male genitalia of the Mexican species *Ae*. *shannoni* Vargas and Downs and the South American species *Ae*. *euiris* Dyar, *Ae*. *milleri* Dyar, *Ae*. *scutellalbum* Boshell-Manrique, and *Ae*. *bejaranoi* Martínez, Carcavillo & Prosen were similar to those of species in the Scapularis Group. He chose not to include any of these species in the Scapularis Group, because of significant differences in the adults and larvae or because the immatures of these species were unknown to him. Although *Ae*. *amateuri* has conspicuously banded tarsi, we assign it to the Scapularis Group without hesitation because that characteristic appears to be the only significant difference from other members of the group.

Females of *Ae*. *amateuri* have the following characteristics of the Scapularis Group presented by Arnell [15]: (1) pleural scales white, not silver, (2) paratergite without scales, (3) subspiracular scale-patch present, (4) hypostigial scale-patch absent, and (5) all wing scales dark. Females of *Ae*. *amateuri* are distinguished from other species of the Scapularis Group most conspicuously by (1) the presence of white tarsal bands, the hindtarsus having white scales at both the base and apex of the tarsomeres and tarsomere 5 entirely white-scaled (Fig 3D), and also by (2) the shape of the subspiracular scale-patch, which has a prominent dorsal extension toward the postpronotum (Fig 3D). *Aedes thelcter* Dyar appears to be the only other species in the Scapularis Group with a dorsal extension to the subspiracular scale-patch, but in that species the extension is not as pronounced. *Aedes amateuri* is very similar to *Ae*. *shannoni* Vargas and Downs, the most notorious differences between these species are found in the adult female: the abdominal terga of *Ae*. *amateuri* have basal bands, whereas in *Ae*. *shannoni* they have small triangular patches on segments 5–6, and the coloration of pleural integument is light brown or golden in *Ae*. *amateuri*, whereas is dark-brown or almost black in *Ae*. *shannoni*. The color pattern of the scales on the scutum of *Ae*. *amateuri* is similar to that of the South American *Ae*. *crinifer* (Theobald), and *Ae*. *amateuri* runs to that species in Arnell’s identification key if the tarsal bands are ignored.

The male genitalia of *Ae*. *amateuri* are characterized by (1) a bluntly conical basal tergomesal lobe of the gonocoxite that bears 1 very strong, bent, and recurved differentiated seta dorsally, about 20 weaker setae, and 3 or 4 slightly longer and stronger setae along its ventral margin, (2) a weakly developed apical tergomesal lobe of the gonocoxite (Fig 4A), and (3) a simple claspette filament without an outer angle, retrorse process, or elongate spicules. The genitalia appear to be inseparable from those specimens of *Ae*. *scapularis* (Rondani) and *Ae*. *shannoni* that lack a retrorse process on the claspette filament.

Larvae of *Ae*. *amateuri* possess all the characteristics considered to be diagnostic of the Scapularis Group by Arnell [15], namely (1) spiculose thoracic and abdominal integument, (2) seta 13-VI short, multiple, and similar to 13-II, (3) comb scales more than 15, in small patch of 2 or 3 irregular rows, (4) saddle complete, and (5) anal papillae without large, darkly pigmented tracheae. Among species of the Scapularis Group, larvae of *Ae*. *amateuri* may be distinguished from those of most species by the combination of (1) head setae 5,6-C single (Fig 4E), (2) spicules of thoracic integument numerous, weakly pigmented, length 4–6 times basal diameter, (3) thoracic seta 3-P single, (4) lateral abdominal seta 6-I,II double and 6-III–VI single, (5) abdominal seta 13-III strongly developed, long, single, (6) comb scales evenly fringed, the median spinule not enlarged, and (7) pecten spines evenly spaced, in straight row to mid-lenght of siphon (Fig 4F). The larvae appear to be indistinguishable from those of *Ae*. *euplocampus* Dyar and Knab.

In keys to North American *Aedes* in the subgenus *Ochlerotatus* [16–18], females of *Ae*. *amateuri* key to *Ae*. *canadensis* (Theobald), but they differ from that species in numerous ways, perhaps most significantly by lacking complete rows of acrostichal and dorsocentral setae on the scutum and lacking scales on the paratergite and laterotergite. These species also differ in scutal ornamentation: in *Ae*. *amateuri* the lateral margins of the scutum are clothed with dark reddish-brown scales that are the darkest scales on the scutum, whereas in *Ae*. *canadensis* those scales are matte white to lustrous white, yellowish-white, pale yellow, or pale golden and are among the palest scales on the scutum. In these same publications, larvae of *Ae*. *amateuri* key to species of the Scapularis Group, either to *Ae*. *scapularis* or *Ae*. *tortilis* (Theobald) in Carpenter and LaCasse [16] depending upon how the density and pigmentation of the cuticular spicules are interpreted; to *Ae*. *trivittatus* (Coquillett), the only species of the Scapularis Group in Canada, in Wood et al. [17]; and to *Ae*. *scapularis* in Darsie and Ward [18].

*Aedes amateuri* appears as the undescribed species no. 1 in the previously pubished study of mosquitoes from Tamaulipas state [19].

Bionomics. Immature stages of *Ae*. *amateuri* were collected from a small pond with colored water but without aquatic vegetation in association with *Ae*. *trivittatus* and a *Culex* species. Adult females were collected in Nuevo León biting collectors at dusk in association with *Ae*. *trivittatus*, *Ae*. *amabilis*, *Ae*. *brelandi/Ae*. *triseriatus*, *Ps*. *ferox*, *Cs*. *particeps*, and *Ur*. *coatzacoalcos*; biting in Tamaulipas state during daytime in association with *Ae*. *trivittatus*, *Ae*. *brelandi*, and *Hg*. *equinus*; and biting at dusk with *Ae*. *trivittatus*, *Ae*. *brelandi*, *Hg*. *equinus*, and *Ps*. *cyanescens*. Since females of *Ae*. *amateuri* can be persistent biters of humans, the species may be involved in the transmission of pathogens.

**Fig 5:**
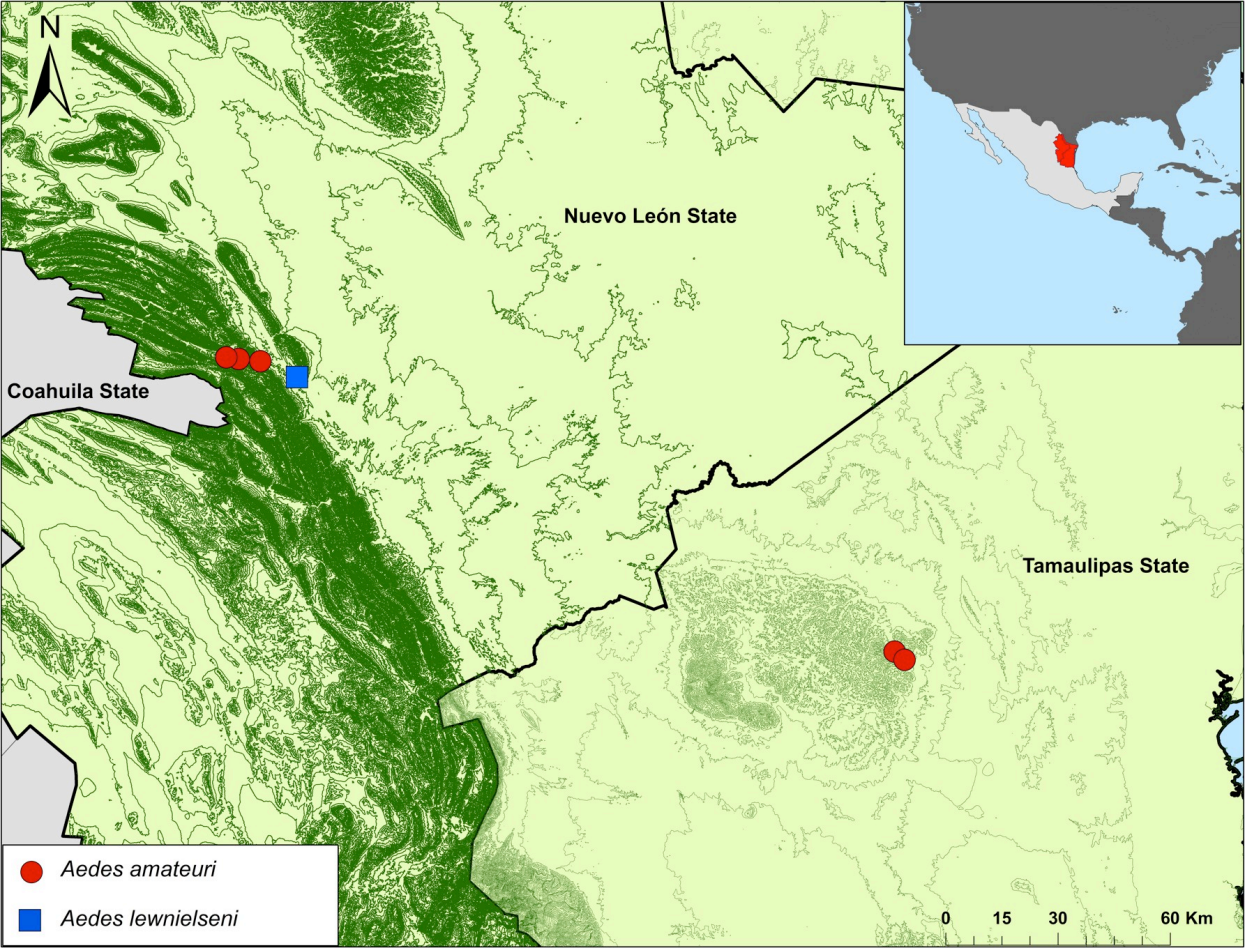
Distribution of *Ae*. *amateuri* n. sp. and *Ae*. *lewnielseni* n. sp.

Distribution (Fig 5). *Aedes amateuri* has been collected in the northeastern Mexican states of Nuevo León (El Manzano and Cienega de González, both locations in Santiago County); and Tamaulipas (El Milagro and Sierra de San Carlos, both locations in Cruillas County). All locations where the species was collected belong to the Grand Folded Sierra of the Sierra Madre Oriental in Nuevo León, and the Sierra de San Carlos in the Coastal Plain of North Gulf in Tamaulipas. *Aedes amateuri* may occur in the forested regions of northeastern Mexico. Since the immature stages develop in ground pools, the species may be distributed in other regions to the north and northwest, to the states of Coahuila, Chihuahua in Mexico, and in the U.S.A.

Etymology. This species is named *amateuri* because it was collected by personnel that were inexperienced in mosquito collections. “Amateur” is a word derived from the Latin “*amatum*,” which means someone who does an activity for love without being professional.

#### *Aedes* (*Protomacleaya*) *lewnielseni* Ortega and Zavortink n. sp. AC1CF45C-89F0-4ACB-AC1B-31EE405DB06A

Types: *Holotype*: Adult ♀ with associated pupal exuviae (Pe) [CC-UL, 243, 08191008-CC], Sierra la Camotera, Santiago, Nuevo León, Mexico (25° 20′ 4′′ N 100° 4′ 22′′ W), elevation above 1855 m, immature stages collected from large tree hole in living oak tree, 19 Oct 2008, col. A. Hernández-Velázquez, A.I. Ortega-Morales, D.A. González-Villareal, J.A. Díaz-López, J. de la Cruz-Zavala, J.J. Hernández-Rodríguez, R. Altunar-López. *Paratypes* (same data as holotype): 4♀, 1 adult ♂ [CC-UL], 2♀ [Bohart Museum], 1♂ [Bohart Museum], 5A♂-Pe and genitalia (G♂) (239, 244, 250, 251, 257, 277, 278, 279, 280, 281) [CC-UL], 22 ♀-Pe (240–243, 245–249, 252–256, 258–265) [CC-UL], 6 Pe (216–221) [CC-UL], 5 larval exuviae (Le) with dead pupae (DP) (223–225, 237–238) [CC-UL], 3 dead larvae (Dl) (226, 231, 234) [CC-UL], 4 DP (236) [CC-UL], 1G♂, 1Pe, 2 Dl [Bohart Museum]; 2A♀, [Bohart Museum, 07191008-CC], (same data as holotype), biting collectors.

**Fig 6:**
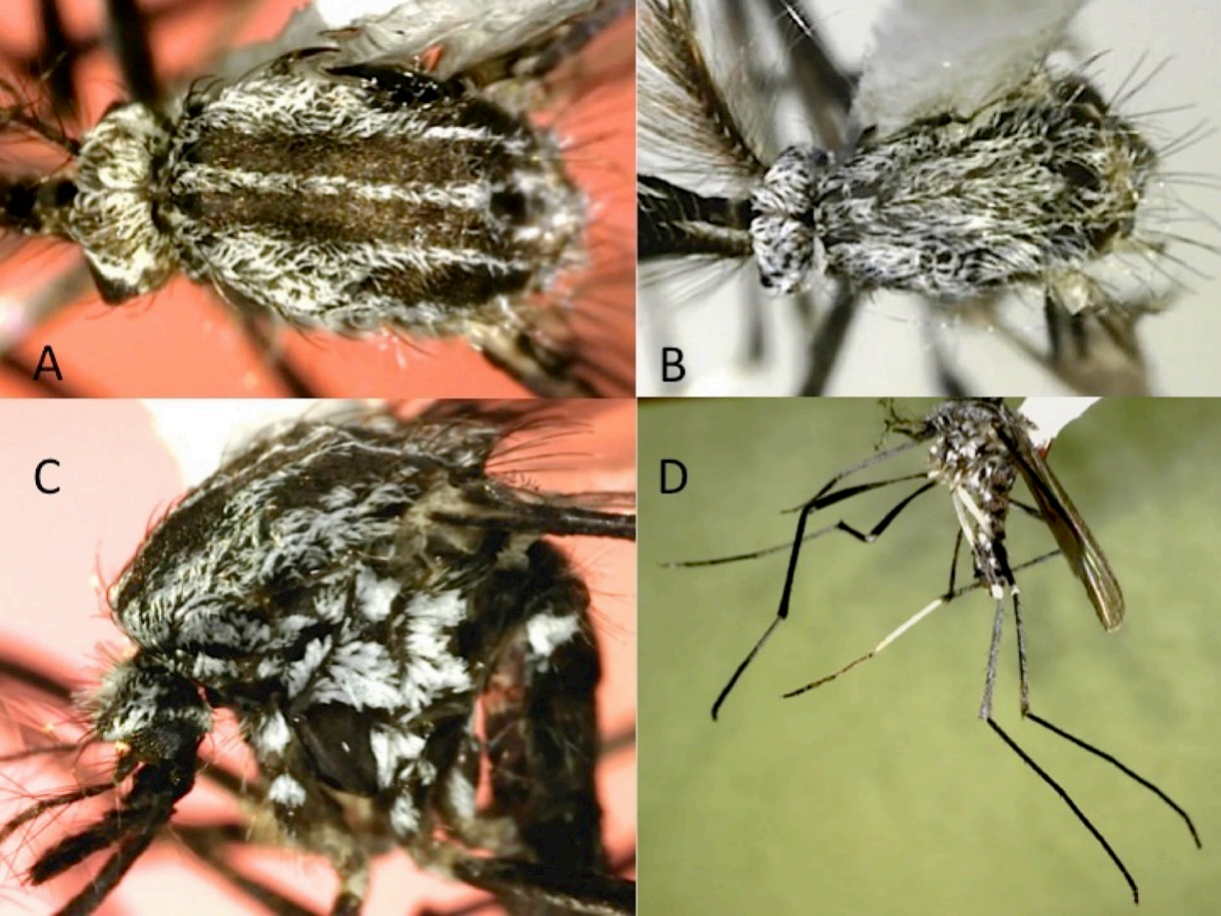
Adult of *Aedes* (*Protomacleaya*) *lewnielseni*. A: Dorsal view of the scutum (female). B: Dorsal view of the scutum (male). C: Lateral view of thorax (female). D: Lateral view of body (female).

Female. Wing about 4.30–4.60 mm; proboscis about 2.89–2.98 mm; forefemur about 2.64–2.76 mm; abdomen about 3.1–3.4 mm. Integument dark brown. *Head*: Erect scales slender, dingy white or tinged with tan, lateral ones sometimes brown; midline and upper eye margin with white narrow curved scales; area laterad of midline with white and black narrow curved scales; side of head with broad white and tan or brown flat scales. Proboscis and palpus black-scaled; palpus 0.20–0.24 length of proboscis. Pedicel brown medially, yellow laterally; flagellomere 1 swollen, with dark scales. *Thorax* (Fig 6A): Acrostichal and dorsocentral setae numerous, in complete rows, humeral, lateral prescutal and posterior fossal setae few. Scutal scales narrow curved, brown, with white scales in acrostichal line, at sides of prescutellar space, and in very broad lateral line from anterior promontory to wing base that extends mesad to or nearly to dorsocentral setae in fossal area and is continued posteriorly as outer dorsocentral line; dark-scaled areas without scattered light scales. Scutellum with long setae and narrow curved scales on all lobes, scales of midlobe white, sometimes brown laterally. Paratergite with large patch of broad white flat scales; antepronotum with setae and white scales; postpronotum with vertical row of setae posteriorly and complete covering of mostly appressed broad, flat white scales, the upper anterior ones dense, sometimes slightly narrowed and curved. Pleura with setae on postspiracular area, prealar knob, proepisternum, upper and posterior mesokatepisternum and upper mesepimeron; white broad, flat, scales in conspicuous patches on postspiracular, hypostigial and subspiracular areas, proepisternum, metameron, in 3 patches on mesokatepisternum, and in large patch on mesepimeron; lower proepisternal scales (prosternal scales of Zavortink [20]) absent or few in an oblique line (Fig 6C and 6D). *Wing*: Completely dark-scaled or with a few white scales near base of costa. *Halter*: White or white- and brown-scaled. *Legs*: Predominately dark-scaled, white scales on posterior surface of fore- and midfemora, on basal 0.5–0.6 of hindfemur, in large knee spots on mid- and hindleg, on underside of foretarsomere 1, and on basal 0.8–0.9 of midtarsomere 1; hindtarsus dark-scaled. *Abdomen*: Tergum I with long setae, a few brown and white scales dorsally, and large patch of white broad flat scales on laterotergite; terga and sterna II–VII dark-scaled with basolateral white patches, the terga sometimes with additional basal white scales.

Male. Essentially as in female except for sexual characters and scutal ornamentation. *Head*: Palpus slightly shorter than proboscis, apex downturned, with numerous long setae from apex of palpomere 3 distad. *Thorax*: Scutum entirely white-scaled (Fig 6B). *Wing*: Base of vein R entirely dark-scaled. *Legs*: Hindtarsomere 1 entirely dark-scaled.

**Fig 7:**
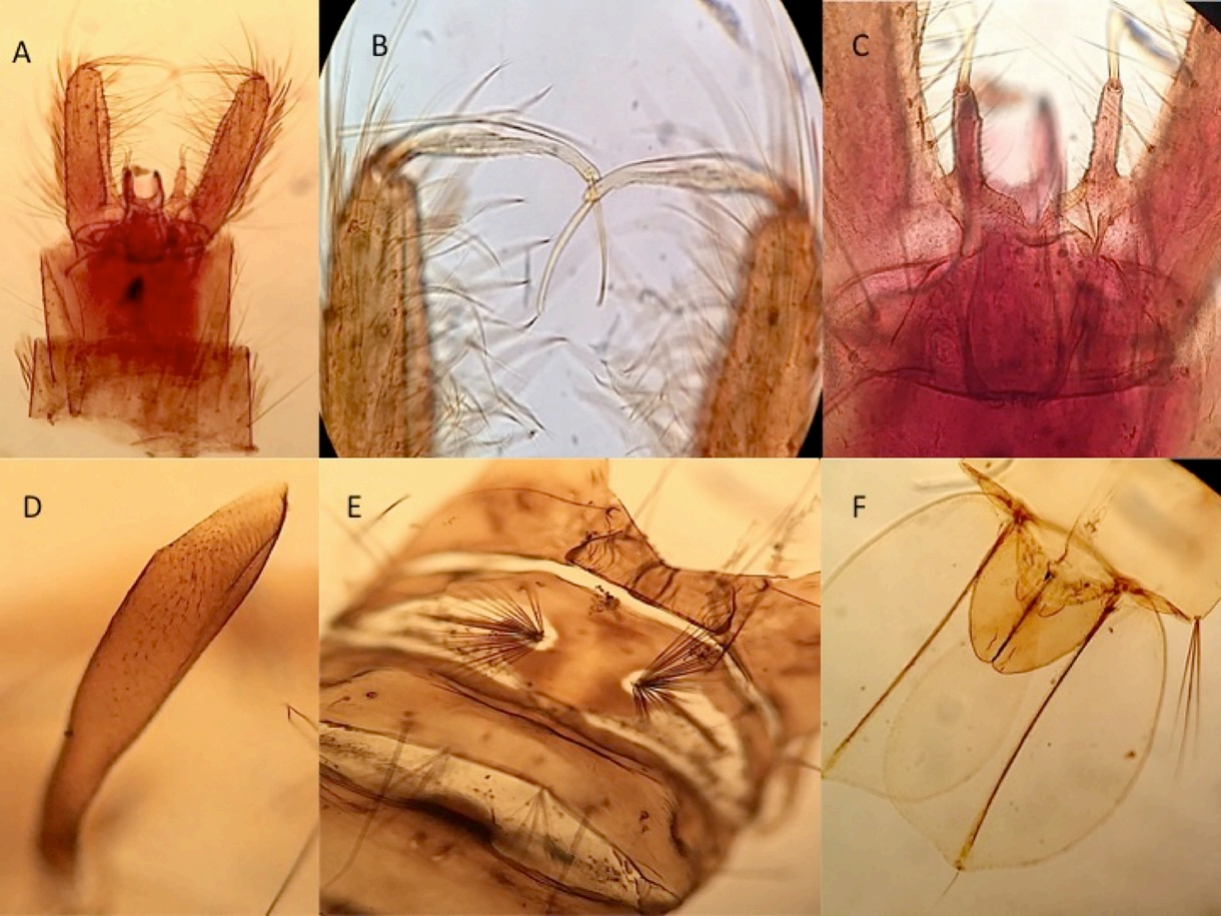
Male genitalia and pupa of *Aedes* (*Protomacleaya*) *lewnielseni*. A: General aspect of genitalia. B: Gonostylus and gonostylar claw. C: Claspette. D: Trumpet. E: Metanotum and seta 1-I. F: Paddle with genital lobe (male).

Male genitalia (Fig 7A–7C). As described for the Kompi Group by Zavortink [20] and apparently indistinguishable from other species. *Gonocoxite*: Basal tergomesal area not swollen, with 10–12 slender setae; median sternomesal sclerite very weakly developed; median sternomesal tuft very weakly developed, the setae few and not strongly curved dorsad. *Phallosome*: Aedeagus relatively short, length 1.5–1.8 times greatest width.

**Fig 8:**
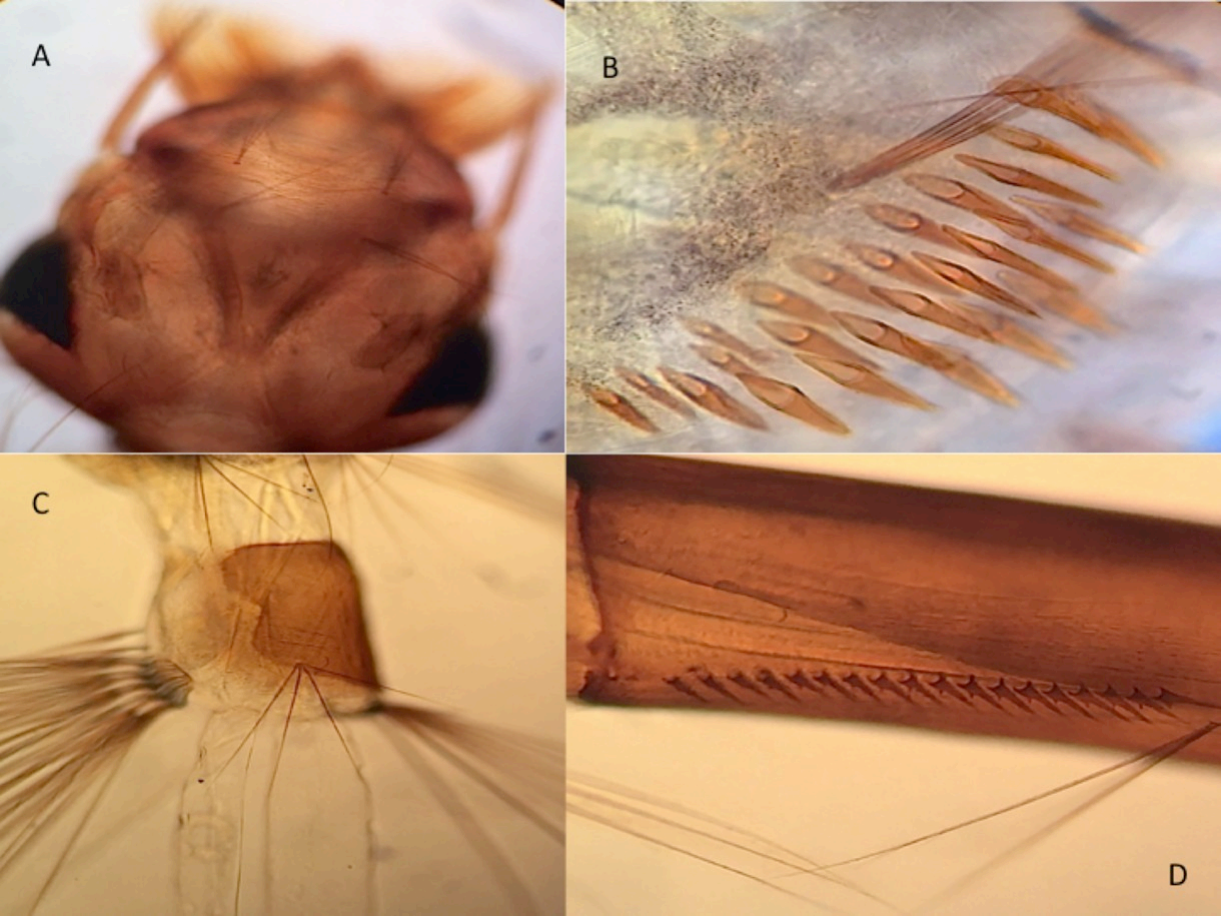
Larva of *Aedes* (*Protomacleaya*) *lewnielseni*. A: Head (dorsal view). B: Comb scales. C: Segment X. D: Pecten of siphon.

Larva. Head: about 1.08–1.12 mm. Siphon: about 1.07–1.10 mm. Saddle: about 0.31–0.39 mm. *Head* (Fig 8A): As broad as long to slightly broader than long; brown, collar darker. Labrum more or less evenly rounded in dorsal aspect. Seta 1-C stout, arising on or near front edge of labrum. Seta 4-C weakly developed, with 2, 3 short branches, much closer to seta 6-C than to midline. Seta 5-C single to triple. Seta 6-C double or triple. Seta 7-C with 2–6 branches. Seta 14-C single. Seta 15-C short, double or triple. Dorsomentum triangular, with 10, 11 teeth on each side of median tooth. *Antenna*: Short, 0.4 length of head; brown; shaft with a few inconspicuous spicules. Seta 1-A single, extending to or nearly to apex of antenna. *Thorax*: Epidermal cells bearing brownish pigment granules. Setae 1,5-P double or triple. Seta 1-M with 3–6 branches, the branches wide-spreading. *Abdomen*: Epidermal cells bearing brownish pigment granules. Seta 1 with branches tapering, wide-spreading; seta 1-I with 4 branches; seta 1-II with 2–4 branches; seta 1-III–VI usually double or triple (single to triple); seta 1-VII strongly developed, long, double. Seta 2-I double or triple; seta 2-II–VI strong, single. Seta 5-I–VI usually with 3–5 branches (3–6), the branches tapering, wide-spreading. Seta 6-I–II with 2–4 branches; seta 6-III–VI double. Seta 12–1 present. Seta with 13 branches tapering, wide-spreading; seta 13-II–V with 3–5 branches; seta 13-VI with 7–9 branches, the branches much shorter and finer than those of 13-V; seta 13-VII usually triple (single to triple). *Segment VIII*: Branches of seta 1-VIII usually finer than those of seta 1-X. Comb with 26–37 scales in patch of 2, 3 irregular rows, each scale long, tapered, evenly fringed (Fig 8B). *Anal segment*: Saddle incomplete, extending 0.6–0.7 down lateral surface of segment. Seta 1-X arising on and slighty shorter than saddle, with 3, 4 branches. Seta 2-X strongly developed, with 3–6 branches. Seta 3-X strongly developed, long, single. Ventral brush (seta 4-X) with 6 pairs of setae (5 pair on grid, 1 pair precratal); seta 4a-X long, at least 2.5 length of saddle; setae with 4 branches, 4c-X usually with 3, 4 branches (2–4). Anal papillae slender, tapered distally; dorsal and ventral papillae subequal in length, 2.4–3.0 length of saddle (Fig 8C). *Siphon*: Brown with darker basal band and lighter apex; index 3.6–4.0; acus distinct, attached. Pecten with 17–18 evenly spaced spines, each with 1 large and several small basal denticles (Fig 8D). Seta 1-S arising beyond pecten, triple.

Pupa. Abdomen: about 3.4 mm. Trumpet: about 0.68 mm. Paddle: about 0.98 mm. With typical countershading, parts dorsal in resting position light brown, parts ventral in resting position straw-colored. Setae in general well pigmented and easily seen. *Cephalothorax*: Seta 4-C strongly developed, single or 2-forked; seta 8-C stronger than seta 9-C, usually single (single to 2-forked); seta 10-C moderately to strongly developed, subequal to or stronger and longer than 12-C. *Trumpet* (Fig 7D): Brown basally, becoming lighter apically. *Abdomen*: Seta 1-I strongly developed, longest branches extending nearly to lateral margin of segment, primary branches simple or with few secondary branches; seta 1-II moderately strong, with 4, 5 branches, the branches finer than primary branches of seta 1-I and sometimes forked (Fig 7E); seta 1-III–V weakly to moderately developed, usually double (single to 4-forked). Seta 2-IV–V inserted mesad of seta 1 and caudad of level of seta 4 of corresponding segment; seta 2-VI inserted mesad or laterad of seta 1-VI and cephalad or caudad of level of seta 4-VI. Seta 5-IV–VI strongly developed, extending to or beyond apex of following segment, 5-IV single or double, 5-V–VI single. Seta 9-VII moderately developed, double; 9-VIII strongly developed, with 3, 4 branches. *Paddle*: Index 1.4–1.5 (Fig 7F).

Systematics. *Aedes* (*Protomacleaya*) *lewnielseni* is a member of the Kompi Group as described and treated by Zavortink [20]. Females of *Ae*. *lewnielseni* are distinguished from all six previously described species of the group by having a conspicuous hypostigial patch of white scales (Fig 6C). Males are distinguished by the combination of the following characteristics: (1) scutum entirely white-scaled (Fig 6B), (2) palpus slightly shorter than proboscis, (3) base of hindtarsomere 1 entirely dark-scaled, and (4) remigium of wing entirely dark-scaled. Larvae are distinguished by the combination of (1) the double or triple head seta 6-C (Fig 8A); (2) the attenuate branches of setae 1,13-I–VI; (3) the strong, single seta 2-II–VI; and (4) the long seta 4a-X of the ventral brush (Fig 8C).

In their general habitus females of *Ae*. *lewnielseni* are most similar to *Ae*. *burgeri* Zavortink, agreeing with that species in having a very broad lateral marginal line of white scales on the scutum that extends mesad to the inner margin of the fossa and in lacking scattered light scales in the dark-scaled areas of the scutum. The six species of the Kompi Group known to Zavortink [20] appeared to belong to three groups of two species each. *Aedes lewnielseni* cannot be allied with any of those groups, suggesting that they have no lasting taxonomic value.

Bionomics. Immature stages of *Ae*. *lewnielseni* were collected from a large tree hole in a living oak tree (*Quercus* sp.). The immature stages were associated with *Ae*. *quadrivittatus* and *Cx*. *thriambus*. Adult females were collected biting collectors in association with *Ae*. *quadrivittatus* and *Ae*. *muelleri*. The possible medical importance is unknown.

Distribution (Fig 5). *Aedes lewnielseni* has only been found in the Sierra la Camotera, Santiago County, Nuevo León, northeastern Mexico, but may occur in the Grand Folded Sierra of the Sierra Madre Oriental of Coahuila, Nuevo León, and Tamaulipas.

Etymology. This species is dedicated to Lewis T. Nielsen (1920–2014), in recognition of his contributions to the field of mosquito taxonomy, both through the training of students and through his own research on the mosquito faunas of Utah and other western regions, and the ecology and distribution of tree hole and snow pool mosquitoes of the western United States. Lew was, additionally, a friend, colleague, and mentor of one of us (TJZ).

## Discussion

Based on our collection records and the known distributions of the mosquito species collected in Nuevo León, three groups of species are recognized. The species of each group have similar geographical distributions, reach their northern or southern distributional limits in the state, and the immature stages of groups 2 and 3 share the same kind of aquatic habitat. The broader distribution of all mosquito species collected in Nuevo León state is summarized in the Table 3.

**Table 3:** Distribution of the mosquito fauna of Nuevo León state.

Species	DIS	Species	DIS	Species	DIS
*An*. *crucians*	NEO	*Ae*. *lewnielseni*	END	*Cx*. *quinquefasciatus*	PNT
*An*. *eiseni*	NEO	*Ae*. *triseriatus*	MEX	*Cx*. *restuans*	MEX
*An*. *franciscanus*	MEX	*Ae*. *zoosophus*	MEX	*Cx*. *salinarius*	MEX
*An*. *pseudopunctipennis*	NEO	*Ae*. *aegypti*	PNT	*Cx*. *stigmatosoma*	NEO
*An*. *punctipennis*	MEX	*Ae*. *albopictus*	PNT	*Cx*. *tarsalis*	MEX
*An*. *quadrimaculatus*	MEX	*Hg*. *equinus*	NEO	*Cx*. *thriambus*	NEO
*An*. *albimanus*	NEO	*Ps*. *columbiae*	C-M	*Cx*. *erraticus*	MEX
*Ae*. *vexans*	HOL	*Ps*. *discolor*	MEX	*Cx*. *arizonensis*	MEX
*Ae*. *epactius*	NEO	*Ps*. *signipennis*	MEX	*Lt*. *bigoti*	NEO
*Ae*. *quadrivittatus*	NEO	*Ps*. *cyanescens*	NEO	*Cs*. *melanura*	MEX
*Ae*. *muelleri*	MEX	*Ps*. *ferox*	NEO	*Cs*. *inornata*	MEX
*Ae*. *amateuri*	END	*Ps*. *ciliata*	NEO	*Cs*. *particeps*	C-M
*Ae*. *bimaculatus*	NEO	*Ps*. *cilipes*	NEO	*Ma*. *dyari*	NEO
*Ae*. *dorsalis*	HOL	*Ps*. *howardii*	NEO	*Ma*. *titillans*	NEO
*Ae*. *dupreei*	NEO	*Cx*. *restrictor*	NEO	*Or*. *alba*	MEX
*Ae*. *nigromaculis*	MEX	*Cx*. *bidens*	NEO	*Or*. *kummi*	NEO
*Ae*. *scapularis*	NEO	*Cx*. *coronator*	NEO	*Wyeomyia* n. sp.	END
*Ae*. *sollicitans*	C-M	*Cx*.*chidesteri*	NEO	*Tx*. *mocetzuma*	NEO
*Ae*. *taeniorhynchus*	NEO	*Cx*. *declarator*	NEO	*Ur*. *syntheta*	MEX
*Ae*. *trivittatus*	MEX	*Cx*. *erythrothorax*	NEO	*Ur*. *coatzacoalcos*	NEO
*Ae*. *amabilis*	END	*Cx*. *interrogator*	NEO	*Ur*. *lowii*	NEO
*Ae*. *brelandi*	MEX	*Cx*. *nigripalpus*	NEO		

CSP: Cosmopolitan; PNT: Pantropical; C-M: Caribbean-Mexico-USA; END: Endemic for Mexico; MEX: Mexico (Overlapping Nearctic and Neotropical regions); NEO: Neotropical; HOL: Holarctic.

### GROUP 1 (Fig 9)

Species that occur in the Nearctic Region that extend into northeastern Mexico where they reach their southern limit of distribution in Nuevo León includes *Culiseta melanura*. This develops in ground pools and extends from the Nearctic Region into the Grand Northamerican Plains in the state of Nuevo León.

**Fig 9:**
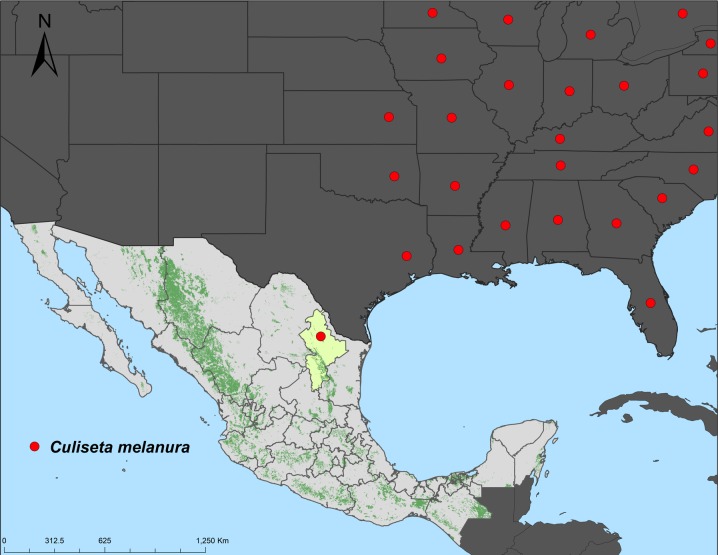
Ground pool inhabiting species that occur in the Nearctic Region and northeastern Mexico.

#### GROUP 2 (Fig 10)

The immature stages of the species that comprise this group inhabit in phytotelmata and artificial containers in tropical forests in the Neotropical Region and extend into northeastern Mexico where most reach their northern limit in Nuevo León and Tamaulipas states: *Anopheles eiseni*, *Ae*. *quadrivittatus*, *Ae*. *amabilis*, *Hg*. *equinus*, and *Cx*. *restrictor*. All members of this group occur in the Grand Folded Sierra of the Sierra Madre Oriental; these mountains comprise part of the Tropical Mesoamerican Corridor, through which all species of this group spread from farther south into northeastern Mexico. “Cumbres de Monterrey” National Park, where wooded habitats and natural regions are conserved, is located in the mountain ranges of the Grand Folded Sierra in Nuevo León. In this region, epiphytic bromeliads, where species such as *Ae*. *quadrivittatus* develop, grow on oaks and other trees; and arboricolous species such as *An*. *eiseni*, *Ae*. *amabilis*, *Hg*. *equinus*, and *Cx*. *restrictor* develop in tree holes that are filled with rain water. While most of these species reach their northernmost range of distribution in northeastern Mexico, *Hg*. *equinus* spreads into Cameron County in Texas [18, 21–22].

**Fig 10:**
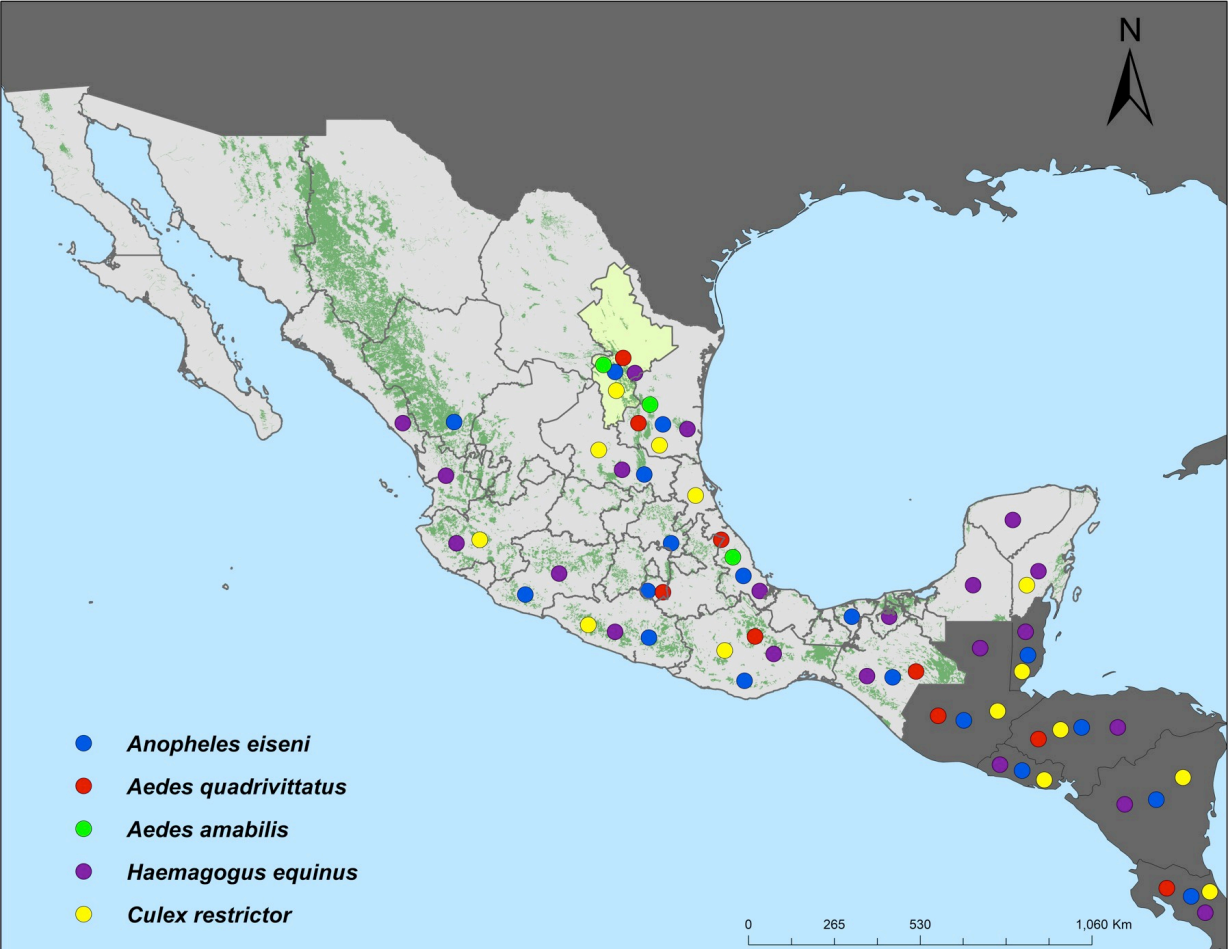
Phytotelmatic and artificial container inhabiting species that spread from the Neotropical Region into northeastern Mexico.

#### GROUP 3 (Fig 11)

Ground pool inhabiting species that extend from the Neotropical Region into northeastern Mexico: *Psorophora cilipes*, *Cx*. *bidens*, and *Ur*. *coatzacoalcos*. These species usually develop in brightly lit bodies of water with abundant floating and emergent aquatic vegetation. They have spread into Nuevo León from humid, wet, swampy regions southeast of Tamaulipas and Veracruz states. *Psorophora cilipes* was originally reported in Mexico by Dyar [23]. Vargas [24] reported the species in Mexico State, but later, in the early revision by Díaz-Nájera and Vargas [25], the species was deleted from the list of Mexican species. Considering that this species was collected only once in a single site, we suppose that *Ps*. *cilipes* is not well established in Nuevo León state or in northeastern Mexico. *Culex bidens* has been reported a few times by the CAIM collectors. This species could be common, developing in swamps in suburban regions of several states of Mexico [26], but the species has not been recorded as far as north as the United States. *Uranotaenia coatzacoalcos* extends from northern South America through southern Mexico into Nuevo León state where it reaches its northernmost limit.

**Fig 11:**
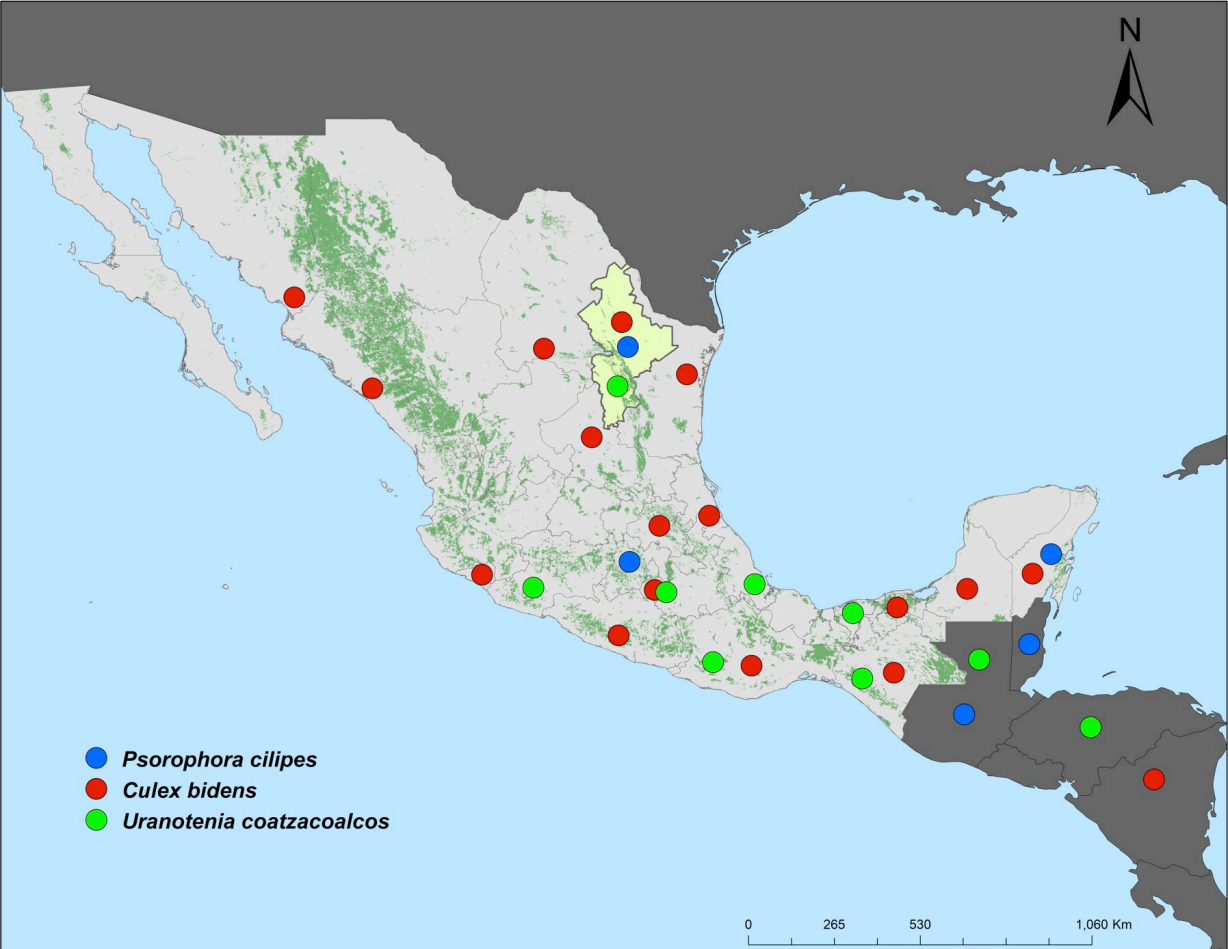
Pond, swamp, and marsh inhabiting species that spread from the Neotropical Region to northeastern Mexico.

#### Species and names deleted from the Nuevo León mosquito fauna

Some species reported to occur in Nuevo León state in earlier publications are not considered to occur in the state in this study. In this section, the species deleted from the Nuevo León fauna and the reasons for the deletions are given, organized by authors of the articles in which the species were reported. The reasons for deletions of *Ae*. *atropalpus* (Coquillett), *Cx*. *peus* Speiser, and *Cx*. *reevesi* Wirth [25]; *Cx*. *pipiens* L. [1]; and *Cx*. *virgultus* Theobald [24] are explained in a previous paper on the mosquitoes of Tamaulipas state [19].

*Psorophora virescens* Dyar and Knab [24]. This species was synonymized with *Ps*. *howardii* Coquillett by Dyar [27].

*Toxorhynchites rutilus* (Coquillett) [1]. Currently, the taxonomy of the *Toxorhynchites* species that occurs in northeastern Mexico is uncertain. *Toxorhynchites rutilus* occurs in southeastern Canada and the eastern United States, extending as far as Texas and possibly northeastern Mexico, where it may occur in symaptry with *Tx*. *moctezuma* in the Nearctic-Neotropical overlapping areas. Since at this point, we cannot confirm how the populations of both species are distributed, we consider this record to be based on misidentified specimens of *Tx*. *moctezuma*.

*Toxorhynchites theobaldi* (Dyar and Knab) [25]. This species is commonly reported in early records of *Toxorhynchites* species from Mexico. Since the resurrection of *Tx*. *moctezuma* and *Tx*. *hypoptes* (Knab) from synonymy with *Tx*. *theobaldi* [12], the distribution of *Tx*. *theobaldi* is uncertain, but it does not occur in Middle America or Mexico. Mexican records of *Tx*. *theobaldi* are most likely based on specimens of *Tx*. *moctezuma*.

*Deinocerites cancer* Theobald [28]. Voucher specimens for this record were found in the IAIM collection. Immature stages were collected from tree holes, mounted on microscope slides and misidentified as *De*. *cancer*. We reviewed those microscope slides and identified the specimens as *Cx*. *restrictor*.

Misidentifications of mosquitoes deposited in IAIM collection; the corroborated species is included in parenthesis

*Anopheles perplexens* Ludlow (*An*. *punctipennis* (Say)); *Aedes melanimon* Dyar (*Culex coronator* Dyar and Knab); *Ae*. *togoi* (Theobald) (*Ae*. *epactius* Dyar and Knab); *Wyeomyia smithii* (Coquillett). (*Wyeomyia* n. sp.).

#### Species from adjacent regions that may occur in Nuevo León

Some species of mosquitoes that have not yet been reported from Nuevo León occur in adjacent areas and may occur within the state. Included among these are eight species that have been recorded in the Lower Rio Grande Valley of Texas and Tamaulipas state: *Ae*. *mitchellae* (Dyar), *Ae*. *thelcter* Dyar, *Cx*. *abominator* Dyar and Knab, *Cx*. *territans* Walker, *Cx*. *corniger* Theobald, *Ps*. *discolor* (Coquillett), *Ps*. *mexicana* (Bellardi), and *Ur*. *sapphirina* (Osten Sacken) [18–19, 29].

#### Medical importance of the mosquitoes of Nuevo León

Some pathogens transmitted by mosquitoes have received great attention in recent years from public health authorities and society due to the arboviral diseases recently new recorded in Mexico such as chikungunya in 2014 and Zika in 2015, both of which caused epidemic outbreaks throughout the country. Currently, dengue fever and Zika are the most important diseases transmitted by mosquitoes due to the large number of cases in humans reported by the Secretariat of Health: 17,795 cases of dengue in 2016 and 9,232 cases of Zika since 2015 to July 2017 [30]. The causal agents of both diseases are transmitted by the bites of infected females of *Ae*. *aegypti* and/or *Ae*. *albopictus*, species that are well established in urban and suburban areas of Nuevo León.

In recent years, West Nile Virus (WNV) has been recognized in birds, horses, and humans in Nuevo León state [31–32]. Some vectors of WNV, such as *Cx*. *quinquefasciatus* and *Cx*. *tarsalis*, have wide distributions in Nuevo León and their populations are usually very high during the rainy season. Adults of *Cx*. *tarsalis* usually stay in dark places in houses during the winter season and become active at the beginning of spring. Other WNV vectors such as *Ae*. *dorsalis*, *Cx*. *nigripalpus*, *Cx*. *restuans*, and *Cs*. *inornata*, have restricted distributions in Nuevo León, develop only small population sizes, and are found only during the rainy season in the summer.

Some arboviruses that cause encephalitis, such as the Venezuelan Equine Encephalitis virus (VEEV), Eastern and Western Equine Encephalitis viruses (EEEV) (WEEV), St. Luis Encephalitis virus (SLEV), and LaCrosse Encephalitis virus (LCV) have not been reported recently or ever in Nuevo León. This does not mean these viruses are absent from the state because those and other arboviruses could be circulating actively in zoonotic cycles in wild areas of Nuevo León. Legislation for surveillance of diseases caused by vector-borne pathogens in Mexico is limited to the search and diagnosis of a few diseases, such as dengue fever, chikungunya, Zika, West Nile fever, and malaria [33–34], neglecting diseases caused by other pathogens that are surely circulating in Mexico, including Nuevo León state. Some vectors of encephalitis viruses occurring in Nuevo León are: (VEEV) *Ae*. *scapularis*, *Ae*. *taeniorhynchus*, *Ps*. *ferox*, *Ps*. *columbiae*, *Ma*. *titillans*; (EEEV) *Ae*. *taeniorhynchus*, *Cx*. *nigripalpus*, *Cs*. *melanura*; (WEEV) *Ae*. *dorsalis*, *Cx*. *quinquefasciatus*, *Cx*. *tarsalis*, *Cs*. *inornata*; (SLEV) *Cx*. *nigripalpus*, *Cx*. *quinquefasciatus*, *Cx*. *restuans*, *Cx*. *tarsalis*; (LCV) *Ae*. *triseriatus*.

In recent years, cases of yellow fever have been reported in some South American countries [35]. This disease occurs in two forms: urban yellow fever for which the virus is transmitted to humans by biting females of *Ae*. *aegypti* and *Ae*. *albopictus*, and sylvan yellow fever for which the virus is transmitted to humans by biting females of *Sabethes* and *Haemagogus* species. In Nuevo León, *Ae*. *aegypti* and *Ae*. *albopictus* are well established in urban and suburban areas, whereas *Hg*. *equinus* spreads through the woodlands of the Coastal Plains of North Gulf. The presence of these species represents an epidemiological risk for yellow fever, in case of this disease is reported in Mexico.

Although there have been no reports of cases of malaria in recent years in Nuevo León, vectors of *Plasmodium* species that cause human malaria occur in the state: *An*. *pseudopunctipennis* and *An*. *albimanus* are the main vectors of *Plasmodium* in the tropical regions of Mexico, whereas *An*. *quadrimaculatus* has been involved as a malaria vector in the United States. A summary of the species of mosquitoes of medical and veterinary importance in Nuevo León and the diseases caused by the pathogens that they transmit is given in Table 4.

**Table 4:** Medical and veterinary importance of the mosquitoes of Nuevo León state.

Species	DENV	ZIKV	CHKV	WNV	YFV	VEEV	EEEV	WEEV	SLEV	LCEV	CEV	TV	Malaria	*D*. *immitis*
*An*. *crucians*													X	
*An*. *pseudopunctipennis*													X	
*An*. *punctipennis*													X	
*An*. *quadrimaculatus*													X	X
*An*. *albimanus*													X	
*Ae*. *vexans*				X				X	X					X
*Ae*. *dorsalis*				X				X						
*Ae*. *scapularis*					X	X								
*Ae*. *sollicitans*														X
*Ae*. *taeniorhynchus*						X	X							X
*Ae*. *trivittatus*												X		X
*Ae*. *triseriatus*										X				X
*Ae*. *aegypti*	X	X	X		X									
*Ae*. *albopictus*	X	X	X		X									
*Hg*. *equinus*					X									
*Ps*. *ferox*						X								
*Ps*. *columbiae*						X								
*Cx*. *nigripalpus*				X			X		X					
*Cx*. *quinquefasciatus*				X				X	X					X
*Cx*. *restuans*				X					X					
*Cx*. *tarsalis*				X				X	X		X			
*Cs*. *melanura*							X							
*Cs*. *inornata*				X				X						
*Ma*. *titillans*						X								

DENV. Dengue fever; ZIKV. Zika; CHIKV. Chikungunya; WNV. West Nile Virus; YFV. Yellow Fever; VEEV. Venezuelan Equine Encephalitis; EEEV. Eastern Equine Encephalitis; WEEV. Western Equine Encephalitis; SLEV. St. Louis Encephalitis; LCEV. LaCrosse Encephalitis; CEV. California Encephalitis Virus; TV. Trivittatus Virus; Malaria; *Dirofilaria immitis* (dog heartworm).
